# Electronic Health Record Population Health Management for Chronic Kidney Disease Care: A Cluster Randomized Clinical Trial

**DOI:** 10.1001/jamainternmed.2024.0708

**Published:** 2024-04-15

**Authors:** Manisha Jhamb, Melanie R. Weltman, Susan M. Devaraj, Linda-Marie Ustaris Lavenburg, Zhuoheng Han, Alaa A. Alghwiri, Gary S. Fischer, Bruce L. Rollman, Thomas D. Nolin, Jonathan G. Yabes

**Affiliations:** 1 Renal-Electrolyte Division, Department of Medicine, University of Pittsburgh School of Medicine, Pittsburgh, Pennsylvania; 2 Department of Pharmacy and Therapeutics, University of Pittsburgh School of Pharmacy, Pittsburgh, Pennsylvania; 3 Division of General Internal Medicine, Department of Medicine, University of Pittsburgh, Pittsburgh, Pennsylvania; 4 Center for Behavioral Health, Media, and Technology, University of Pittsburgh School of Medicine, Pittsburgh, Pennsylvania; 5 Center for Research on Heath Care, Division of General Internal Medicine, Department of Medicine and Biostatistics, University of Pittsburgh, Pittsburgh, Pennsylvania

## Abstract

**Importance:**

Large gaps in clinical care in patients with chronic kidney disease (CKD) lead to poor outcomes.

**Objective:**

To compare the effectiveness of an electronic health record–based population health management intervention vs usual care for reducing CKD progression and improving evidence-based care in high-risk CKD.

**Design, Setting, and Participants:**

The Kidney Coordinated Health Management Partnership (Kidney CHAMP) was a pragmatic cluster randomized clinical trial conducted between May 2019 and July 2022 in 101 primary care practices in Western Pennsylvania. It included patients aged 18 to 85 years with an estimated glomerular filtration rate (eGFR) of less than 60 mL/min/1.73m^2^ with high risk of CKD progression and no outpatient nephrology encounter within the previous 12 months.

**Interventions:**

Multifaceted intervention for CKD comanagement with primary care clinicians included a nephrology electronic consultation, pharmacist-led medication management, and CKD education for patients. The usual care group received CKD care from primary care clinicians as usual.

**Main Outcomes and Measures:**

The primary outcome was time to 40% or greater reduction in eGFR or end-stage kidney disease.

**Results:**

Among 1596 patients (754 intervention [47.2%]; 842 control [52.8%]) with a mean (SD) age of 74 (9) years, 928 (58%) were female, 127 (8%) were Black, 9 (0.6%) were Hispanic, and the mean (SD) estimated glomerular filtration rate was 36.8 (7.9) mL/min/1.73m^2^. Over a median follow-up of 17.0 months, there was no significant difference in rate of primary outcome between the 2 arms (adjusted hazard ratio, 0.96; 95% CI, 0.67-1.38; *P* = .82). Angiotensin-converting enzyme inhibitor/angiotensin receptor blocker exposure was more frequent in intervention arm compared with the control group (rate ratio, 1.21; 95% CI, 1.02-1.43). There was no difference in the secondary outcomes of hypertension control and exposure to unsafe medications or adverse events between the arms. Several COVID-19–related issues contributed to null findings in the study.

**Conclusion and Relevance:**

In this study, among patients with moderate-risk to high-risk CKD, a multifaceted electronic health record–based population health management intervention resulted in more exposure days to angiotensin-converting enzyme inhibitors/angiotensin receptor blockers but did not reduce risk of CKD progression or hypertension control vs usual care.

**Trial Registration:**

ClinicalTrials.gov Identifier: NCT03832595

## Key Points

**Question:**

Does a multidisciplinary team-based approach across a large health system using electronic health records and population health management strategy reduce progression of chronic kidney disease (CKD) and improve evidence-based care among patients with high-risk kidney disease?

**Findings:**

In this cluster randomized clinical trial that included 1596 patients with chronic kidney disease with high risk of progression to kidney failure who were not seeing a nephrologist, a multifaceted intervention vs usual care control did not reduce risk of CKD progression, but rather increased exposure to angiotensin-converting enzyme inhibitors/angiotensin 2 receptor blockers over a median follow-up of 17 months.

**Meaning:**

The results of this randomized clinical trial indicate that an electronic health records and population health management strategy using a multifaceted intervention addressed many of the implementation barriers to evidence-based care delivery but did not reduce CKD progression.

## Teaser

This cluster randomized clinical trial examines the effectiveness of an electronic health record–based population health management intervention vs usual care for reducing chronic kidney disease progression.

## Introduction

Large gaps between optimal guideline-concordant care and current clinical practice for chronic kidney disease (CKD) management exist that are associated with poor clinical outcomes, high health care costs, and racial inequities in kidney health.^1,2,3^ These gaps are likely to widen, as the burden for managing the growing and complex CKD population falls on primary care clinicians (PCCs), including physicians and advanced practice clinicians (APCs), due to a dearth of nephrologists.^4^ PCCs report limited CKD knowledge, time constraints, complex case-mix, competing acute illness priorities, and inadequate system-based resources to address these gaps in CKD care.^5,6,7,8^ With emerging CKD management guidelines and newer therapeutics, there is a need to prioritize population-level strategies that enable rapid implementation of evidence-based care in a scalable and equitable way, while shifting the focus of CKD management to value-based care.^9,10,11^

Electronic health record (EHR) tools can be leveraged to identify, risk stratify, and deliver timely clinical decision support (CDS) to improve delivery of guideline-based CKD care.^12,13,14,15,16^ Use of EHR-based population health management (PHM) is a potentially high-effect, low-cost, scalable intervention that can standardize CKD management across a health system and improve efficiency of resource allocation. Prior studies using individual-level strategies, such as CDS,^17,18,19,20,21^ pharmacist support,^22,23^ and nephrology guidance,^24^ had mixed success in improving quality of care. This may be partly due to the lack of multifaceted intervention at the health system, clinician, and patient level. As recommended by the American Diabetes Association and Kidney Disease Improving Global Outcomes, a multidisciplinary team-based care model is needed to improve implementation of optimal CKD care.^25^

We conducted a pragmatic, cluster randomized clinical trial, the Kidney Coordinated Health Management Partnership (Kidney CHAMP), to test whether a multifaceted EHR-based PHM intervention would reduce the likelihood of progression of kidney disease and improve evidence-based care compared with usual care in patients with high-risk CKD not presently seeing a nephrologist.

## Methods

### Study Overview

The Kidney-CHAMP study was conducted in 101 University of Pittsburgh Medical Center (UPMC)–affiliated primary care practices across Western Pennsylvania. The study protocol was published previously^26^ (Supplement 1, Supplement 2, and Supplement 3) and approved by the University of Pittsburgh institutional review board and quality improvement committee. Results were reported using the Consolidated Standards of Reporting Trials (CONSORT) reporting guidelines.

### Eligibility

Patients aged 18 to 85 years with an estimated glomerular filtration rate (eGFR) less than 60 mL/min/1.73m^2^, who were not seeing a nephrologist, and with high risk of CKD progression were screened from May 2019 to Nov 2021. High-risk CKD was defined as eGFR of 15 to 29 mL/min/1.73m^2^, 5-year risk of end-stage kidney disease of 4% or greater determined using a validated 4-variable kidney failure risk equation (KFRE),^27^ or based on an internal machine learning–based risk prediction model incorporating rapid decline in eGFR. In November 2019, the eligibility criteria were broadened to include all eligible patients regardless of their insurance status due to fewer than anticipated eligible patients with just UPMC Health Plan insurance. Patients with a baseline eGFR less than 15 mL/min/1.73m^2^, who were receiving maintenance dialysis, or who had undergone kidney transplant were excluded. The date of enrollment was the date of first PCC (physician or APC) visit after eligibility screening, and those without a PCC visit within 1 year of screening were excluded.

### Trial Procedures

PCC practices were 1:1 randomized as clusters to intervention or usual care to minimize contamination between arms and stratified by their estimated number of eligible patients at baseline by the study biostatistician using a computer-generated software. Given the cluster randomization, there was no allocation concealment for PCCs or patients. Intervention practices received regular, individual, on-site, or virtual outreach to ensure PCC engagement and academic detailing. The EHR-integrated PHM dashboard was reviewed monthly to identify eligible patients. All eligible patients in the intervention arm were sent a letter by mail introducing the program and provided with an opportunity to opt out by calling the study nurse. If neither the PCC nor the patient opted out, then implied consent was assumed, and the patient enrolled.

### Intervention

The multifaceted intervention bundle included timely nephrology guidance, pharmacist-led medication management, and CKD education. Targeted automated electronic consultations provided concise and actionable guideline-based recommendations for reducing cardiovascular and CKD progression and improving medication safety.^28,29^ The targeted automated electronic consultations were also used to educate PCPs by providing rationale of recommendations and benefits and safety of newer heart/kidney medications.

Pharmacists completed a comprehensive medication review and assessed overall safety, ease of use, and the affordability of the medication regimen. After discussions with the multidisciplinary team of a nephrologist, pharmacist, and physician extender, individualized recommendations were routed to the PCC’s EHR inbox and placed in the patient’s health record within 1 week of their upcoming appointment and sent to other specialists if relevant to facilitate care coordination. During the appointment, a real-time CDS reminded the PCC to review recommendations, place orders, and refer for CKD education. Within 1 month after the PCC visit, nurse educators used telemedicine to provide personalized education to patients using standardized materials and addressed kidney replacement therapy options, including medical management without dialysis as appropriate.^30^ Due to poor rates of PCC referral for education, an opt out approach was adopted starting January 1, 2021. During follow-up, electronic consultations, medication reviews, and education sessions were conducted every 4 to 6 months. Intervention fidelity was maintained by standardized training on updated guidelines, direct observation, and random health record audits. Patients enrolled from practices randomized to usual care continued to receive CKD care from PCCs. The planned recruitment period of 18 months and average follow-up of 24 months was modified due to COVID-19 pandemic–related delays in enrollment, resulting in extension of the recruitment period to 30 months and change in follow-up duration until the primary outcome was achieved or until the end of intervention period (July 31, 2022).

### Outcomes and Data Collection

The primary outcome was time from first PCC visit to 40% or greater reduction in eGFR or ESKD defined as an eGFR of 10 mL/min/1.73m^2^ or less or initiation of kidney replacement therapy, including maintenance dialysis or kidney transplant. Secondary outcomes included hypertension control, ACEi/ARB use, and exposures to potentially unsafe medications (nonsteroidal anti-inflammatory drugs, glyburide, metformin, or gemfibrozil if eGFR<30 mL/min/1.73m^2^). Adverse events included hyperkalemia, hospitalizations, emergency department visits, and mortality. Exploratory outcomes included use of statins, sodium-glucose cotransporter-2 inhibitors, glucagon-like protein-1 receptor agonists, and effect of intervention on medication therapy problems.

Routinely collected patient-level EHR data were abstracted and supplemented by manual health record abstraction as needed. Publicly available PCC practice-level data were obtained. To determine baseline eGFR, we averaged the 2 most recent eGFRs (calculated using the race-free Chronic Kidney Disease Epidemiology Collaboration [CKD-EPI] 2021 equation) that were 90 days or more apart within 3 years before the PCC visit. Urine albumin-to-creatinine ratio (UACR) was calculated using urine albumin or protein quantification or estimated using a urine dipstick protein.

### Statistical Analysis

We determined that 1653 patients provided 80% power to detect a hazard ratio (HR) of 0.64, or a 5% absolute risk reduction in intervention arm, assuming a primary end point rate of 15% in the usual care group at 24 months, 20% loss to follow-up, α = .05, and within-practice intraclass correlation of 0.01. All primary analyses were intention-to-treat.^31^ We used discrete-time survival methods to examine the occurrence of the primary end point at 6-month intervals averaging all outpatient eGFRs within a window to account for random eGFR fluctuations and potential ascertainment bias. We used a generalized linear mixed model (GLMM) with random practice intercepts to account for practice-level clustering and fixed effects for intervention and time. Adjusted models included prespecified patient (age, sex, self-reported race, baseline eGFR) and practice size. Hospice or death were treated as competing events, as was medical management without dialysis to limit bias as it may have resulted due to the intervention itself or more accurate diagnosis coding in the intervention arm. Those who did not reach any end point were censored at the end of study. In the secondary analysis, we used eGFR as a continuous variable. Sensitivity analyses included eGFR smoothing-spline mixed-effects models and requiring 2 consecutive eGFR values less than the 40% decline threshold.

For secondary outcomes, we used GLMM to model outpatient blood pressure (BP) averaged at 6-month intervals and analyzed the BP goal as a binary outcome. The models included treatment, time, and treatment by time interaction with random patient and practice intercepts. We analyzed medication exposure days using GLMM with random practice effect controlling for baseline exposure days and adjusted for patient age, sex, race, and practice size.

Prespecified subgroup analyses included assessment of primary outcome stratified by age, sex, CKD stage, diabetes, hypertension control at baseline (<140/90 mm Hg and <130/80 mm Hg), and ACEi/ARB in patients with albuminuria. A post hoc analysis was done to evaluate the effect of the intervention on albuminuria. All statistical analyses were performed in R, version 4.2.1 (R Foundation).

## Results

### Participants

**Figure 1.**
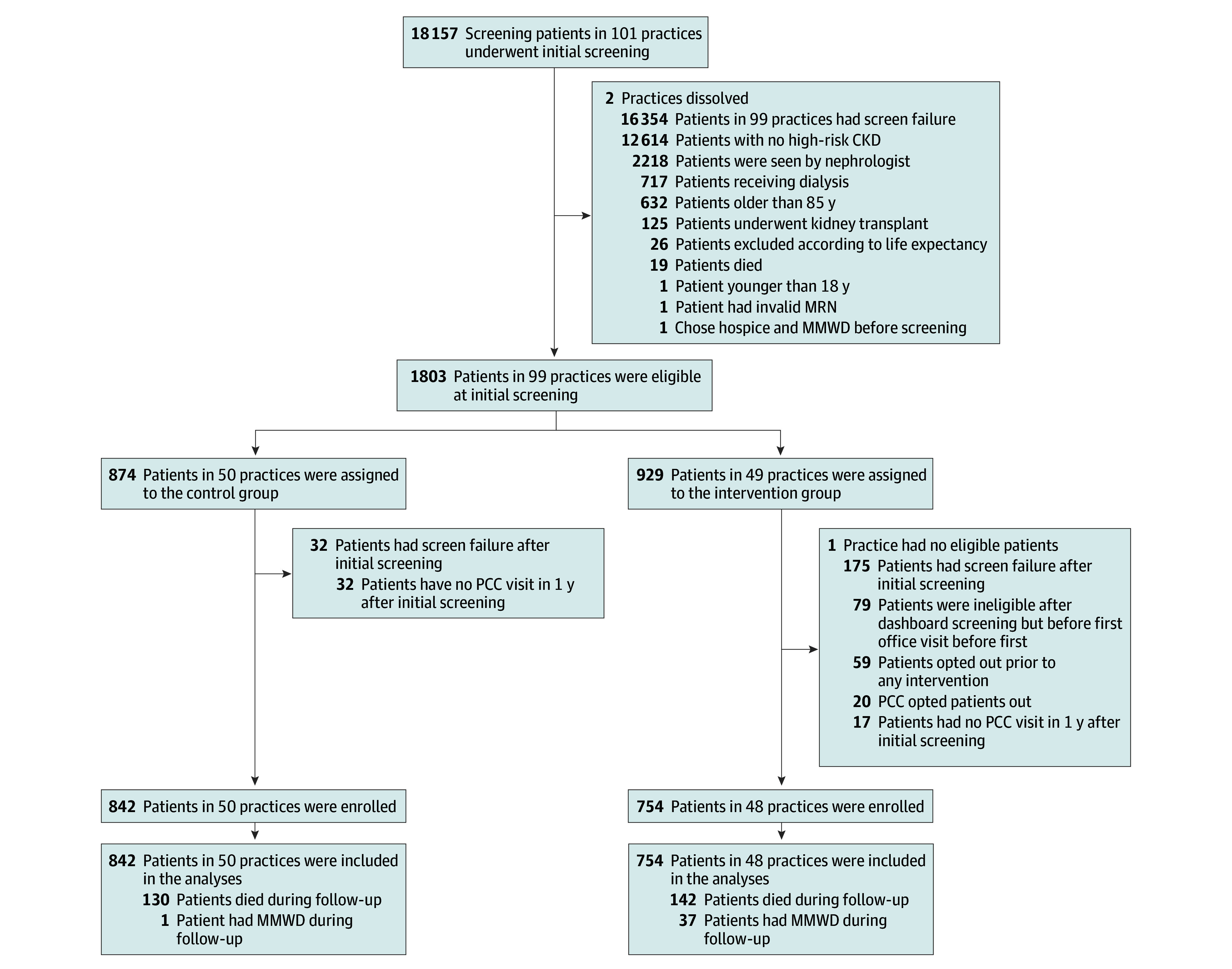
Consort Diagram CKD indicates chronic kidney disease; MRN, medical record number; MMWD, medical management without dialysis; PCC, primary care clinician.

Among 101 primary care study practices, we screened 18 157 patients and identified 1803 potentially eligible study participants. Of these, we included 1596 who met all protocol eligibility criteria and provided implied consent (Figure 1) and enrolled 1317 (83%) after the March 1, 2020, COVID-19 period.

Baseline sociodemographic and clinical characteristics of patients and characteristics of PCC practices were similar between trial arms (Table 1; eTable 1 in Supplement 4). Mean (SD) age was 74 (9) years, 928 (58%) were female, and 127 (8%) were Black. The mean (SD) eGFR was 36.8 (7.9) mL/min/1.73m^2^, and the median (IQR) UACR was 85 (15.1-421.5) mg/g. Although 914 patients (74%) had a 5-year KFRE of 4% or greater at eligibility screening, this changed to 752 (52%) when the CKD-EPI creatinine (2021) equation was used to calculate baseline eGFR. Using a recently suggested risk triage cutoff of a 5-year KFRE of 3% or greater, 927 (64%) had high-risk CKD.^32^

**Table 1.** Baseline Characteristics of Patients by Intervention Arm

Variable	Mean (SD) or No (%)			Absolute standardized bias
	Overall (N = 1596)	Control group (n = 842)	Intervention group (n = 754)	
Age, y	73.6 (8.9)	73.4 (8.8)	73.8 (9.1)	4.17
Female	928 (58)	519 (62)	409 (54)	15.02
Male	668 (42)	323 (38)	345 (46)	15.02
Race				
Black	127 (8.0)	76 (9.05)	51 (6.8)	8.40
White	1449 (91%)	753 (89)	696 (92)	10.00
Other^a^	20 (1.3)	13 (1.5)	7 (0.)	5.57
Hispanic ethnicity^b^	9 (0.6)	5 (0.6)	4 (0.5)	0.88
Married^c^	837 (52%)	426 (51)	411 (55)	7.73
Rural-urban category (RUCA score)^d^				
Metropolitan	1225 (77)	658 (78)	567 (75)	6.97
Micropolitan	280 (18)	161 (19)	119 (16)	8.81
Town/rural	89 (5.6)	22 (2.6)	67 (8.9)	27.22
BMI^e^	32.2 (7.4)	32.1 (7.3)	32.3 (7.6)	2.61
Systolic blood pressure, mm Hg	131.4 (16.9)	131.6 (17.2)	131.1 (16.6)	2.99
Diastolic blood pressure, mm Hg	74.1 (10.6)	74.4 (10.8)	73.87 (10.4)	4.90
Blood pressure <130/80 mm Hg	628 (39)	323 (38)	305 (40)	4.28
Blood pressure <140/90 mm Hg	1118 (70)	587 (70)	531 (70)	1.55
Congestive heart failure	501 (31)	249 (30)	252 (33)	8.29
Diabetes	1027 (64)	546 (65)	481 (64)	NA
Type 1	26 (1.6)	12 (1.4)	14 (1.9)	3.40
Type 2	1001 (62.7)	534 (63.4)	467 (61.9)	3.07
Hypertension	1512 (95)	801 (95)	711 (94)	3.73
Cardiovascular disease	1248 (78)	653 (78)	595 (79)	3.29
Charlson Comorbidity Index score	6.8 (2.9)	6.8 (3.0)	6.8 (2.8)	2.66
Serum creatinine level, mg/dL	1.7 (0.4)	1.7 (0.4)	1.7 (0.4)	0.33
eGFR CKD-EPI, mL/min/1.73m^2^^f^	36.8 (7.9)	36.6 (7.9)	37.1 (7.9)	6.69
Serum potassium level, meq/L	4.4 (0.5)	4.4 (0.5)	4.4 (0.4)	1.27
Hemoglobin, g/dL^g^	12.4 (1.7)	12.3 (1.7)	12.5 (1.7)	9.63
Serum albumin level, g/dL^h^	3.8 (0.4)	3.8 (0.5)	3.8 (0.4)	4.10
Hemoglobin A_1C_ level (only for diabetes), %^i^	7.4 (1.5)	7.4 (1.5)	7.4 (1.5)	0.13
Urine albumin-creatine ratio, mg/g^j^^,^^k^	85.0 (15.1-421.5)	84.6 (14.0-410.3)	86.0 (16.3-434.0)	1.17
2-y KFRE, %^j^	1.4 (0.7-3.1)	1.3 (0.7-3.1)	1.4 (0.8-3.0)	5.22
5-y KFRE, %^j^	4.2 (2.2-9.3)	4.1 (2.0-9.3)	4.2 (2.3-9.1)	5.33
5-y KFRE ≥3%	927 (64)	465 (62)	462 (66)	9.62
CKD stages				
2	10 (0.6)	4 (0.5)	6 (0.8)	4.04
3a	197 (12)	99 (12)	98 (13)	3.76
3b	1110 (70)	590 (70)	520 (69)	2.40
4	277 (17)	147 (17)	130 (17)	0.57
5	2 (0.1)	2 (0.2)	0	6.90
Albuminuria stages				
A1	509 (35)	276 (37)	233 (33)	6.66
A2	483 (33)	245 (32)	238 (34)	3.51
A3	459 (32)	233 (31)	226 (32)	3.27
Medication use at baseline				
NSAID	96 (6.0)	53 (6.3)	43 (5.7)	2.49
ACEi/ARB	733 (46)	391 (46)	342 (45)	2.17
SGLT2i	42 (2.6)	18 (2.1)	24 (3.2)	6.50
GLP1-RA	80 (5.0)	32 (3.8)	48 (6.4)	11.70
Statin (moderate to high intensity)	808 (51)	427 (51)	381 (51)	0.36
No. of hospitalizations or emergency department visits in last 1 y^l^	2.1 (1.7)	2.1 (1.8)	2.1 (1.6)	2.12
No. of PCC visits in last year^m^	3.7 (2.5)	3.6 (2.3)	3.9 (2.6)	10.99

Abbreviations: ACEi, angiotensin-converting enzyme inhibitor; ARB, angiotensin receptor blocker; BMI, body mass index (calculated as weight in kilograms divided by height in meters squared); CKD, chronic kidney disease; CKD-EPI, Chronic Kidney Disease Epidemiology Collaboration; eGFR, estimated glomerular filtration rate; GLP1-RA, glucagon-like peptide-1 receptor agonists; KFRE, kidney failure risk equation; NA not applicable; NSAID, nonsteroidal anti-inflammatory drug; PCC, primary care clinician; RUCA, rural-urban commuting area codes; SGLT2i, sodium-glucose cotransporter-2 inhibitor.

SI conversion factors: to convert albumin to g/L, multiply by 10; for creatinine to μmol/L, multiply by 88.4; for hemoglobin to g/L, multiply by 10; for hemoglobin A_1c_ to the proportion of total hemoglobin, multiply by 0.01; for potassium to mmol/L, multiply by 1.

^a^ Included Asian individuals and those who declined to answer.

^b^ Ethnicity has 8 missing values: 6 in the control group and 2 in the intervention group.

^c^ Marital status had 1 missing value: 1 in the control group.

^d^ RUCA had 2 missing values: 1 in the control group and 1 in the intervention group.

^e^ BMI had 33 missing values: 17 in the control group and 16 in the intervention group.

^f^ Baseline eGFR was determined using average of 2 most recent eGFRs at least 90 days apart within 3 years prior to the PCC visit. These data were available for 1569 patients (98.3%), and 1512 (94.7%) had 2 eGFR values within 1 year prior to the PCP visit. For 15 patients, the 2 available eGFRs were less than 90 days apart and were averaged to calculate baseline eGFR. For 12 patients, only 1 eGFR value was available within the 3-year look-back period, which was used as their baseline eGFR.

^g^ Hemoglobin had 43 missing values: 26 in the control group and 17 in the intervention group.

^h^ Serum albumin had 42 missing values: 23 in the control group and 19 in the intervention group.

^i^ Hemoglobin A_1C_ had 15 missing values: 10 in the control group and 5 in the intervention group.

^j^ Median (first quartile, third quartile).

^k^ Urine albumin-creatine ratio, KFRE 2-year, KFRE 5-year, and albuminuria stage had 145 missing values: 88 in the control group and 57 in the intervention group.

^l^ No hospitalization or emergency department visits in last year were found in 885 patients: 474 in the control group and 411 in the intervention group.

^m^ No PCC visits in the last year were found in 58 patients: 30 in the control group and 28 in the intervention group.

The median follow-up period was 17.0 months (IQR, 12.0-23.0). Attrition from the health system was low; only 40 patients (2.5%) had missing EHR data in last 6 month prior to the study end date. During the follow-up period, the median number of PCC visits per year was 3.2 (IQR, 2.0-4.6), but 79 patients (32 intervention, 47 control) did not have any PCC visit.

### Treatment Fidelity

Electronic consultations and medication management encounters were completed for more than 97% of patients, with a mean of 2.3 to 2.5 encounters per patient (eTable 2 in Supplement 4). Education sessions were completed for 469 patients (62.2%), with an average 1.9 sessions per patient due to low referral by PCCs initially and study team staffing issues. Only 187 patients (11.7%) in the intervention arm and 205 (12.8%) in the control arm were seen by an outpatient nephrologist during the study period.

### Primary End Point

**Figure 2.**
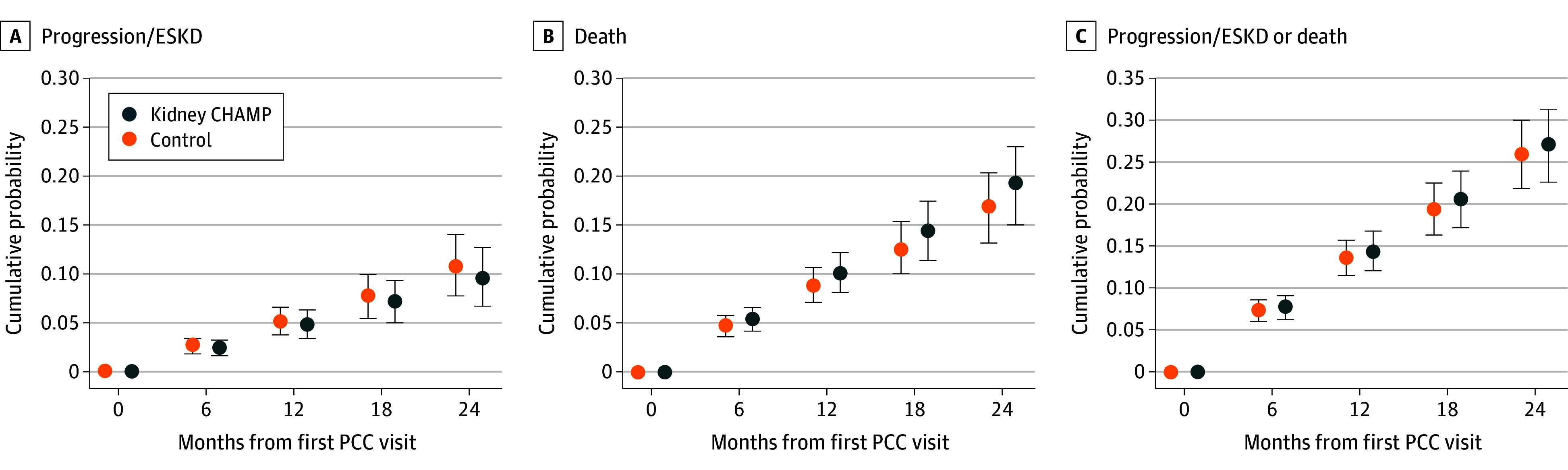
Adjusted Cumulative Event Probability Estimates for Primary Outcome and Survival End Points ESKD indicates end-stage kidney failure; PCC, primary care clinician.

**Figure 3.**
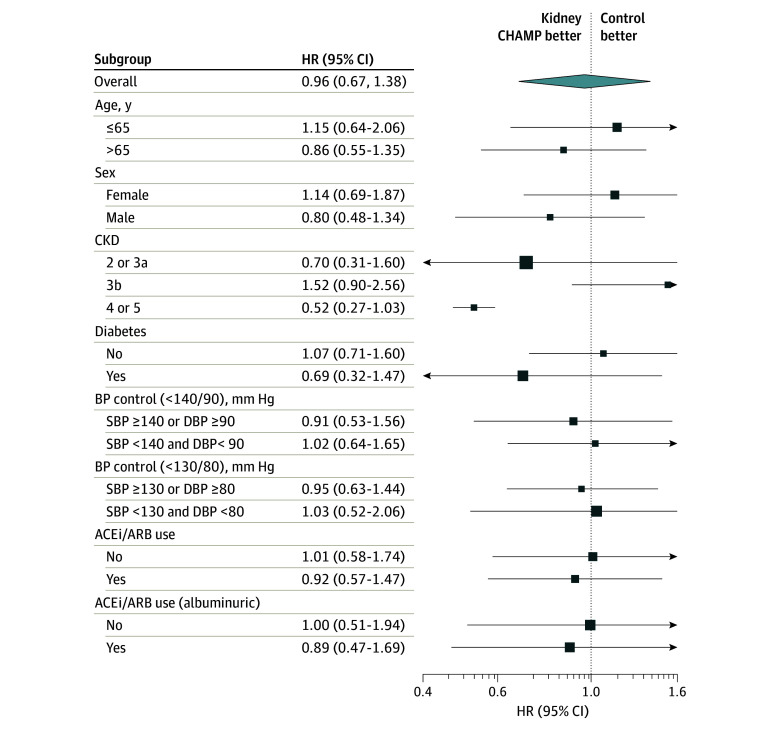
Subgroup Analyses Adjusted Intervention Effect Estimates for Progression/End-Stage Kidney Disease ACEi indicates angiotensin-converting enzyme inhibitor; ARB, angiotensin receptor blocker; BP, blood pressure; CKD, chronic kidney disease; DBP, diastolic blood pressure; HR, hazard ratio; SBP, systolic blood pressure.

There was no significant difference in the rate of primary end point by treatment assignment (57 [7.6%] intervention vs 72 [8.6%] control) even after adjusting for age, sex, race, baseline eGFR, and PCC practice size (HR, 0.96; 95% CI, 0.67-1.38; *P* = .82) (Figure 2). At 24 months, the estimated adjusted percentage of the primary end point was 9.7% (95% CI, 6.6%-12.7%) and 10.9% (95% CI, 7.7%-14.1%) in the intervention and control arm, respectively (Figure 2; Table 2). There was no difference in the primary outcome in subgroups stratified by age, sex, CKD stage, diabetes, hypertension control, or ACEi/ARB use (Figure 3). Similarly, no significant difference in the primary outcome was seen when stratified by race in a post hoc analysis (eTable 3 in Supplement 4). In a secondary analysis using eGFR as a continuous variable, eGFR slopes were similar between the arms (Table 2).

**Table 2.** Primary and Secondary End Points Intervention Effect Estimates

End point	Estimate (95% CI)		Kidney CHAMP vs control, hazards ratio, slope difference, or rate ratio estimate (95% CI)	*P* value^a^
	Kidney CHAMP	Control		
Primary outcome				
≥40% Reduction in eGFR ESKD, cumulative % at 24 mo^b^	9.7 (6.6 to 12.7)	10.9 (7.7 to 14.1)	0.96 (0.67 to 1.38)	.82
Secondary outcomes				
Death, cumulative % at 24 mo^b^	19.2 (15.1 to 23.1)	16.9 (13.2 to 20.4)	1.09 (0.84 to 1.41)	NA
≥40% Reduction in eGFR/ESKD or death, cumulative % at 24 mo^b^	27.1 (22.6 to 31.3)	26.0 (21.8 to 29.9)	1.05 (0.85 to 1.30)	
Confirmed progression^c^/ESKD, cumulative % at 24 mo^b^	5.9 (4.2 to 7.7)	6.1 (4.4 to 7.7)	0.94 (0.58 to 1.52)	
eGFR, mL/min/1.73m^2^ per y slope^d^	−1.007 (−1.365 to −0.648)	−1.143 (−1.497 to −0.789)	0.136 (−0.368 to 0.640)	
BP^d^				
Systolic BP, mm Hg per mo slope	0.005 (−0.057 to 0.067)	0.087 (0.026 to 0.147)	−0.082 (−0.168 to 0.005)	NA
Diastolic BP, mm Hg per mo slope	−0.039 (−0.075 to −0.003)	−0.006 (−0.041 to 0.030)	−0.033 (−0.084 to 0.017)	
Hypertension control (achieved BP <140/90 mm Hg), log-odds per month slope	0.011 (−0.002 to 0.024)	0.0005 (−0.012 to 0.013)	0.011 (−0.008 to 0.029)	NA
Hypertension control (achieved BP <130/80 mm Hg), log-odds per month slope	0.086 (0.073 to 0.100)	0.079 (0.066 to 0.092)	0.007 (−0.011 to 0.025)	NA
Medications^e^				
ACEi/ARB, exposure days per year rate	196.8 (174.9 to 218.7)	163.1 (146.3 to 179.9)	1.21 (1.02 to 1.43)	NA
ACEi/ARB (in patients with UACR ≥300 mg/g), exposure d per y rate	222.5 (182.9 to 262.1)	177.5 (146.2 to 208.8)	1.25 (0.95 to 1.65)	
NSAID, exposure d per y rate	5.3 (2.6 to 8.0)	6.7 (3.7 to 9.7)	0.80 (0.51 to 1.25)	
Glyburide (in patients with type 2 diabetes), exposure d per y	2.4 (−3.7 to 8.5)	2.0 (−1.6 to 5.7)	1.18 (0.03 to 53.78)	
Metformin (in patients with type 2 diabetes and eGFR <30), exposure d per y	17.8 (−6.5 to 42.2)	16.0 (−1.7 to 33.6)	1.12 (0.12 to 9.96)	

Abbreviations: ACEi, angiotensin-converting enzyme inhibitor; ARB, angiotensin receptor blocker; BP, blood pressure; eGFR, estimated glomerular filtration rate; ESKD, end-stage kidney disease; NA, not applicable; NSAID, nonsteroidal anti-inflammatory drug; UACR, urine albumin-creatine ratio.

^a^ *P* value not reported for secondary outcomes as analyses did not adjust for multiplicity.

^b^ Survival end points: estimates, confidence intervals, and *P* values were derived from discrete-time survival analyses using generalized linear mixed models with complementary log-log link and flexible baseline hazards via restricted cubic splines, random practice intercepts, and fixed effects for intervention group adjusted for age, sex, race, baseline eGFR, and practice size.

^c^ Confirmed progression is defined as having a 40% or greater reduction in eGFR that was maintained in the next outpatient eGFR measurement.

^d^ Continuous eGFR and BP end points: estimates, confidence intervals, and *P* values were derived from linear mixed models with random patient intercepts nested within random practice intercepts, and fixed effects for intervention group, linear time, and group×time interaction as adjusted for age, sex, race, and practice size. Hypertension control end points: estimates, confidence intervals, and *P* values were derived from generalized linear mixed models using logit link and binomial family with random patient intercepts nested within random practice intercepts, and fixed effects for intervention group, linear time, and group×time interaction adjusted for age, sex, race, and practice size.

^e^ Medication end points: estimates, confidence intervals, and *P* values were derived from generalized linear mixed models using log link and negative-binomial family with random practice intercepts, and fixed effects for intervention group adjusted for baseline medication exposure days, age, sex, race, and practice size, and log of the number of follow-up days as offset. Gemfibrozil was excluded from analyses since among those with an eGFR of less than 30 at baseline, active use was found only in 1 patient.

### Secondary End Points

There was no significant difference in hypertension control between the arms (Table 2). ACEi/ARB exposure was 21% more frequent (rate ratio [RR], 1.21; 95% CI, 1.02-1.43) in the intervention (197 days per year; 95% CI, 175-219) compared with control (163 days per year; 95% CI, 146-180). The effect was slightly higher but with wider CIs when restricted to patients with a UACR of 300 mg/g or greater (RR, 1.25; 95% CI, 0.95-1.65). Exposure days per year to nonsteroidal anti-inflammatory drugs, glyburide (in patients with type 2 diabetes), and metformin (in patients with type 2 diabetes and a baseline eGFR <30 mL/min/1.73m^2^) were similar between arms (Table 2). Among the 814 medication review encounters completed by phone during the study period, a drug record discrepancy (ie, difference between patient-reported medication list and EHR medication list) was identified in 599 encounters (73.6%). The estimated mean change in UACR from baseline to 18 months was similar between the arms (intervention vs control mean difference in change, −18.2 mg/g; 95% CI, −117.6 mg/g to 81.2 mg/g; eTable 4 in Supplement 4). In sensitivity analyses, using a smoothing-spline mixed-effects model for eGFR or requiring 2 consecutive eGFR values with more than 40% decline to determine progression yielded similar results as the main analyses (Table 2).

### Adverse Events

A total of 272 participants (17%) died. The hazards of death and the composite of primary outcome and death (Figure 1; Table 2), emergency department visits/hospitalizations, and severe hyperkalemia were all similar between study arms (eTable 5 in Supplement 4).

## Discussion

This pragmatic implementation trial of an EHR-based PHM approach for CKD demonstrated scalability and patient and PCC acceptance and met prespecified rates of participant enrollment and retention. However, we found no difference in the risk of CKD progression in patients with moderate-risk to high-risk CKD who were randomized to either the intervention or usual care groups at a median of 17.0 months’ follow-up, and secondary analyses failed to identify a subgroup for which the intervention was more effective, although intervention arm patients tended to have more exposure days to ACEi/ARB.

The multifaceted intervention overcame implementation barriers at the patient, clinician, and health system level.^10,11^ We minimized burden on busy PCCs and patients by using an EHR-based PHM approach to identify, triage, and leverage telemedicine to promote standardized care pathways across a large academically affiliated health system. We also successfully enrolled patients from 13 counties in Western Pennsylvania, including 23.6% from rural or micropolitan regions, thus facilitating equitable access to specialized care for the fast-growing CKD population. The low PCC and patient opt-out rates suggested acceptance of the PHM approach. Participating primary care practices were also heterogenous in terms of academic affiliation, geographical location, access to specialists, practice size, and use of physician extenders, thus supporting the generalizability of our findings. We incorporated several components to optimize population-level value-based kidney health as identified in a recent systematic review, including automated detection of higher-risk cases, educational support, non–patient-facing nephrologist review, and dynamic integration with existing workflows.^33^ Clinician learning during the process of treating patients was promoted by incorporating education in the electronic consultations. Moreover, the intervention was designed based on pilot work that demonstrated high need and receptiveness of this program among PCCs and incorporated feedback from PCCs and health system and health informatics leadership teams to ensure harmonization with PCC workflow. Lastly, the study team garnered leadership support during the design phase to build a clinician-centric approach, built rapport with community PCCs, and educate them on gaps in CKD care and emerging management guidelines.

The multidisciplinary team-based intervention was designed to provide a comprehensive assessment and management plan for the PCCs to help comanage complex patients. Multidisciplinary care teams in CKD have been associated with a reduced risk of all-cause mortality, hospitalization, and eGFR decline^34^ and are advocated by recent clinical practice guidelines from the Kidney Disease Improving Global Outcomes and American Diabetes Association.^25,35^ We balanced the need to provide multidisciplinary quality care while doing so in a scalable and resource-efficient way by using a central, highly trained clinical team and leveraging telemedicine. For electronic consultations, we used trained APCs instead of nephrologists to conduct a detailed health record review to determine CKD etiology, risk factors, and need for diagnostic workup and formulate an initial management plan based on the latest clinical practice guidelines and document using a standard format. The pharmacist independently completed a comprehensive medication review and addressed kidney and non–kidney-related medication therapy problems, such as drug-drug interactions, affordability, and polypharmacy. The nephrologist’s role was to supervise APCs and pharmacists and devise an individualized plan after case discussion. This approach allowed us to efficiently use nephrologists’ expertise and time. While case discussion with multiple team members may limit scalability in some settings, this was especially important during the study period to avoid conflicting recommendations with rapidly changing guidelines and a need to incorporate the art of medicine when guidance was lacking (eg, SGLT2i use in older adults). Future work is needed to determine how patients can best be triaged to APCs, pharmacists, and/or nephrologist for improving workflow efficiencies.

Past studies for improving CKD care have had mixed success in improving the quality of care or clinical outcomes.^17,18,19,20,21,23,36^ Carroll et al^37^ conducted one of to our knowledge the few large clinical implementation studies comparing CDS alone vs with practice facilitation in more than 6600 patients with CKD who were followed for 2 years. Although the study showed a positive effect on reducing CKD progression, it required a resource-intensive on-site coordinator, and results were limited due to significant dropout and lack of usual care control. Our intervention addressed many of the limitations of prior studies, and design strengths included large sample size, pragmatic implementation design, cluster randomization at the practice level to limit clinician-level bias, comparison with a contemporaneously enrolled usual care arm to control for secular trends, use of a multifaceted and multilevel intervention, focus on important CKD and cardiovascular outcomes, alignment with PCC workflow, provision of education and self-management support for patients, and longitudinal follow-up. By using an opt-out enrollment process for nephrology electronic consultations and CKD education referral, our intervention addressed alert fatigue that is common with CDS that require clinician action and prioritized CKD care. Despite these strengths, we were unable to support the primary hypothesis.

Several factors may have contributed to the null effect in our study, including a shorter follow-up period, unchanged PCC prescribing behavior due to unfamiliarity with new guidelines, lack of CKD quality metrics and incentives for PCCs, therapeutic inertia, pandemic-related issues on workflow, or patients’ inability to afford their medications (particularly SGLT2i) or prioritize their health over social needs. The clinical effect of newer therapeutics (SGLT2i and GLP-1Ra) in our study may have been limited due to implementation later in the study period and for varying indications in accordance with newer evidence and guidelines that came out during the study period. Among the PCCs, there may have been early and late adopters of the PHM care delivery approach for comanaging complex patients with CKD. PCCs are inundated with multiple patient-related tasks, messages, and insufficient time to address overt patient concerns and routine preventive care, which may have interfered with CKD management.^38^ A recent implementation study to improve use of evidence-based therapies in patients seen in cardiology clinics showed positive results.^39^ This may reflect a culture difference in a specialty clinic where the focus is on disease-specific management vs primary care where the focus is on whole patient care. Ongoing evaluation of qualitative determinants of intervention implementation among PCCs and patients will inform future refinements to increase integration and uptake in primary care. Lastly, the effect of the intervention may have been diluted due to enrollment of patients with moderate-risk CKD as a result of a change in eGFR equations (race-based as reported by most laboratories was used for screening) during the study period and a largely older study cohort in whom the risk of CKD progression may have been overestimated by KFRE.^40,41^ Only 25% of the patients were seen by an outpatient nephrologist during the study period, and rates were similar among both groups, making it highly unlikely that this diluted the intervention effect. The nephrology visit rate was comparable with national data^42^ given that only 17% of the patients had CKD stage 4 to 5. Additionally, COVID-19–related effects on health care and social needs and limited nephrology access for one-fourth of the study cohort living outside of metropolitan areas may have resulted in low nephrology visits.

We also faced several unanticipated challenges during the COVID-19 pandemic that potentially shifted the priorities of patients, clinicians, and health systems away from chronic disease management toward acute medical illnesses and emergency vaccination dissemination. Unfortunately, we had limited power to analyze results before and during the COVID-19 period since 83% of the patients were enrolled after March 1, 2020, and even the pre–COVID-19–enrolled patients were likely affected by the challenges. The pandemic worsened demands on PCC time, increased fragmentation of medical and social care, and created siloed access and affordability challenges for health care services and medications. Moreover, focus shifted from research, resulting in delays in PCC site and patient enrollment, requiring us to extend recruitment and shorten the follow-up duration from the planned 24 months to the median 17 months. As a result, our mean intervention touch points were lower than planned for patients enrolled closer to the study end date, and the event rate for the primary outcome was low. A high rate of competing event of death (17%) may have been partly due to the pandemic. Additionally, workflow shifts from in-person to virtual platforms hindered engagement of patients in education sessions and PCCs in outreach meetings. Future studies that enhance a PHM approach by incorporating more frequent follow-up for patients with greater illness severity, enhanced health system resources to address social determinants of health, pharmacist-led medication prescribing to minimize PCC burden, more robust educational and incentivization strategies to modify PCC prescribing behavior, and longer follow-up are needed to evaluate effective and scalable implementation strategies for CKD care, especially to test the effectiveness of newer therapeutics in a real-world setting. Moreover, with rapidly evolving guidelines on indications for use of newer kidney- and heart-protective medications and the designation of cardiovascular-kidney-metabolic syndrome,^43^ future studies that incorporate newer-risk triage tools using genetic, biomarker, and social risk factor data and target patients who are not receiving optimal guideline-concordant care are needed.

### Limitations

Our study should be considered in light of certain limitations. First, medication use was determined by EHR prescription data and may not accurately reflect medication adherence. Second, the bundled intervention limited our ability to examine the individual effects of each component. However, several incremental enhancements in chronic disease processes of care are often needed to affect outcomes.^44,45^ Third, the study was powered to detect a 5% absolute risk reduction at 24 months in the rate of primary outcome in intervention arm, which was deemed a clinically meaningful effect while keeping the total sample size logistically feasible, but may have been an ambitious target. Lastly, our findings may apply only to White patients, who comprised 91% of the study participants.

## Conclusions

This cluster randomized clinical trial found that a multifaceted EHR-based PHM intervention resulted in more exposure days to ACEi/ARB but did not reduce risk of CKD progression or hypertension control vs usual care among patients with moderate-risk to high-risk CKD.

## Notes

Supplement 1. Trial protocol

Supplement 2. Statistical analysis plan

Supplement 3. Grant proposal plan

Supplement 4. eTable 1. Baseline characteristics of PCP practices by intervention arm eTable 2. Treatment fidelity in intervention arm eTable 3. Effect of intervention on primary outcome stratified by race eTable 4. Effect of intervention on albuminuria (using fitted a mixed effects model adjusted for age, sex, race, baseline eGFR, practice size and diabetes) eTable 5. Adverse events by intervention arm

Supplement 5. Data sharing statement
