# Supplementary material for: Electronic Health Record Population Health Management for Chronic Kidney Disease Care: A Cluster Randomized Clinical Trial
Source: JAMA Intern Med. 2024 Apr 15;184(7):737–47. doi: 10.1001/jamainternmed.2024.0708 (PMC11019443; doi:10.1001/jamainternmed.2024.0708)
Supplement: Supplement 1. — Trial protocol [file jamainternmed-e240708-s001.pdf]

Electronic Health Record based Population health management to optimize care in CKD: Kidney **C**oordinated **H**e**A**lth **M**anagement **P**artnership (Kidney CHAMP) trial

Trial registration: ClinicalTrials.gov NCT03832595

Funding Sponsor: 1R01DK116957 (PI, Abdel-Kader & Jhamb)
National Institutes of Diabetes and Digestive and Kidney Diseases
National Institutes of Health
2 Democracy Plaza
6707 Democracy Boulevard
Bethesda, MD 20892-5458

Protocol version 1.0

I. Study Summary

Synopsis

**Title**	Kidney **C**oordinated **H**e**A**lth **M**anagement **P**artnership (Kidney CHAMP) trial
**Short Title**	K CHAMP Trial
**Study Description**	This is a randomized clinical trial to test the effectiveness of a multifaceted EHR-based PHM intervention to improve evidence-based CKD care in high-risk patients
**Objectives**	Primary Objective<br>To perform a 42-month pragmatic, cluster RCT comparing the effect of an EHR-based PHM intervention versus usual care on key processes of care in 1,650 high-risk CKD patients.<br>Aim 1a: To examine the effect of the intervention on systolic blood pressure (SBP) in HTN patients.<br>Aim 1b: To examine the effect of the intervention on RAASi use in albuminuric patients.<br>Aim 1c: To examine the effect of the intervention on exposures to potentially unsafe medications.<br><br>Secondary Objective<br>To test the clinical effectiveness of a multifaceted PHM intervention in reducing kidney disease progression in 1,700 high-risk CKD patients enrolled in a 42-month pragmatic, cluster RCT
**Primary Outcome**	≥40% decline in estimated glomerular filtration rate (eGFR) or end stage kidney disease
**Secondary Outcomes**	• blood pressure control<br>• renin-angiotensin aldosterone system inhibitors use<br>• exposure to potentially unsafe medications
**Study Population**	1,650 high-risk CKD patients not presently seeing a nephrologist
**Phase or Trial Type**	Effectiveness
**Description of Sites/Facilities Enrolling Participants**	100 University of Pittsburgh Medical Center (UPMC)-affiliated PCP practices located across southwest Pennsylvania University of Pittsburgh; University of Pennsylvania
**Description of Study Intervention**	Intervention bundle includes nephrology electronic consults, pharmacist-led medication reviews and nurse-led CKD education<br><br>Usual Care: per PCP as routine

**Study Duration**	42 months
**Participant Duration**	24 months

II. Introduction

CKD is associated with an unacceptably high human and financial cost. Over 12 million US adults have CKD stage 3-5.[6] As the population ages and diabetes (DM), HTN, and obesity rates increase, the prevalence of CKD will grow.[6] Kidney disease is the 9th leading cause of death[48] and attributable Medicare expenditures are $80 billion.[8,19,49] Over 1/3rd of this is spent on ESRD patients, who represent < 5% of patients with CKD.[19]

The overwhelming burden of CKD care falls to PCPs. PCPs deliver most care to patients with non-dialysis dependent CKD due to its growing prevalence and the relative dearth of nephrologists.[10-12,50-52] However, PCPs report that limited CKD knowledge, time constraints, complex case-mix, and inadequate system-based resources contribute to gaps in CKD care.[12,14,15,17,38,51,53] These gaps include poor patient education,[16] inadequate diagnostic evaluation,[12,27,38,54] suboptimal treatment of HTN and use of RAASi in albuminuric patients,[19,23,55-57] inappropriate medications or dosages,[21,58-61] and late referrals of high-risk patients.[13,19,20] These shortcomings inevitably lead to increased CKD progression, hospitalizations, and mortality.[20,25,26,62-64]

Novel system-based interventions are needed. The above observations underscore an urgent need for system-based interventions to improve CKD care and outcomes. We recently conducted a national survey and found the overwhelming majority of PCPs endorse systematic interventions to improve CKD care.[14] One potentially high-impact, low-cost intervention that has improved outcomes in other chronic diseases is EHR- based PHM.[65,66] CKD is an ideal setting to evaluate the impact of EHR-based PHM due to the: 1) high prevalence of disease, 2) ability to detect high-risk disease with widely used biomarkers (i.e., creatinine/eGFR, change in eGFR, urine albuminuria), 3) baseline gaps in care that provide opportunities for improvement, and 4) patient benefit and health system savings conferred by avoiding or delaying catastrophic outcomes (e.g., ESRD).[19,31,67] National primary care organizations have called for the use of EHR-based PHM in primary care[46] and a recent NIDDK conference advocated for urgent research examining the effectiveness of CKD PHM.[68]

A conceptual model. A conceptual model of care[69,70] provides an approach to examine care deficiencies.[71] Disease management consists of 7 simplified steps (**Figure 1**). At each step, the potential for lapses in care exists. Leveraging IT tools to risk stratify patients, deliver decision support, and to provide electronic guidance with specific recommendations overcomes barriers at nearly each step by providing timely cognitive support to aid risk assessment, diagnostic evaluation, and treatment selection while lowering the burden on PCPs.[72,73] However, studies are needed to test the feasibility and effectiveness of these strategies in improving CKD outcomes.

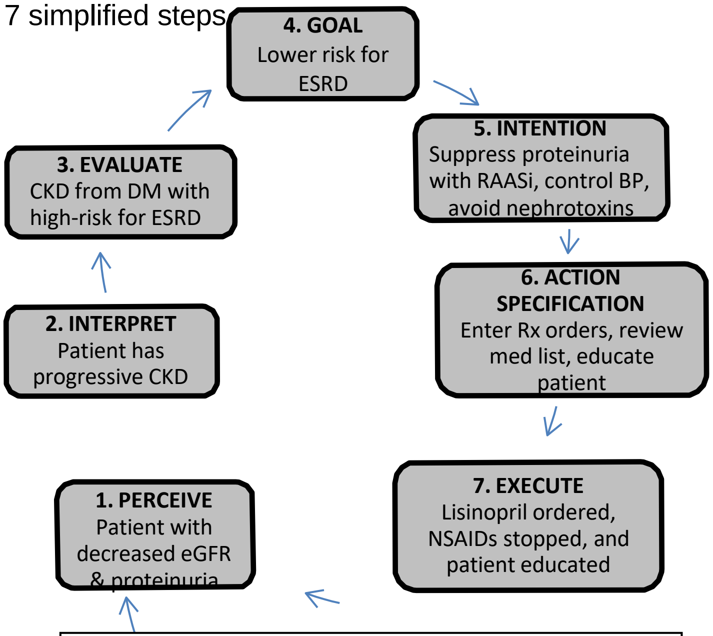

Fig 1. A Conceptual Model for CKD Management

Improving alignment between patient risk and treatment intensity. PCPs struggle to recognize high-risk patients early in their course.[13,14,19] In the US, 2/3rds of incident dialysis patients have less than 1 year of nephrology care before initiating dialysis, leading to greater morbidity and mortality.[13,19,20] However, fewer than 1/3rd of non-dialysis dependent CKD patients with an eGFR< 60 are at high risk for poor outcomes.[74] Given the scarcity of nephrologists, a vital need exists for tools to effectively risk stratify the CKD population and improve the efficiency of resource allocation.[1,76] *This study identifies high-risk patients earlier in their disease course, when outcome trajectories can be improved*.

Actionable strategies to improve CKD care and outcomes in high-risk disease. Key evidence-based process of care targets in CKD are improved: 1) HTN control,[19,44,55,56] 2) RAASi use in proteinuric CKD,[23,57] 3) avoidance of inappropriate medications or dosages,[21,58-61] and 4) timely nephrology referrals in high-risk CKD.[13,19,20] These strategies have been demonstrated to slow CKD progression, prevent ESRD, decrease hospitalizations, and improve patient safety.[13,19-21,23,55-61] Several approaches have been shown to enhance adoption of these critical processes of care: a) electronically delivered expert guidance by nephrologists,[13,77] which slowed CKD progression, b) pharmacist led medication reconciliation,[43,78-80] which decreased medication related problems, and c) patient education,[56,57] which increased patient engagement and self- management.[81,82] However, large pragmatic studies validating the effectiveness of these interventions are lacking.

Summary and Implications: Combining complementary interventions in a *highly* pragmatic, cluster RCT of EHR-based PHM for high-risk CKD patients could establish a novel, exportable strategy to improve patient care, safety, and outcomes (**Figure 2**). Our multifaceted intervention will improve CKD risk stratification, resource allocation, adoption of evidence-based interventions, and patient medication safety. The intervention may thereby improve CKD outcomes and transform approaches to CKD care. Further, the study will deliver templates, algorithms, and code to enable dissemination to other settings.

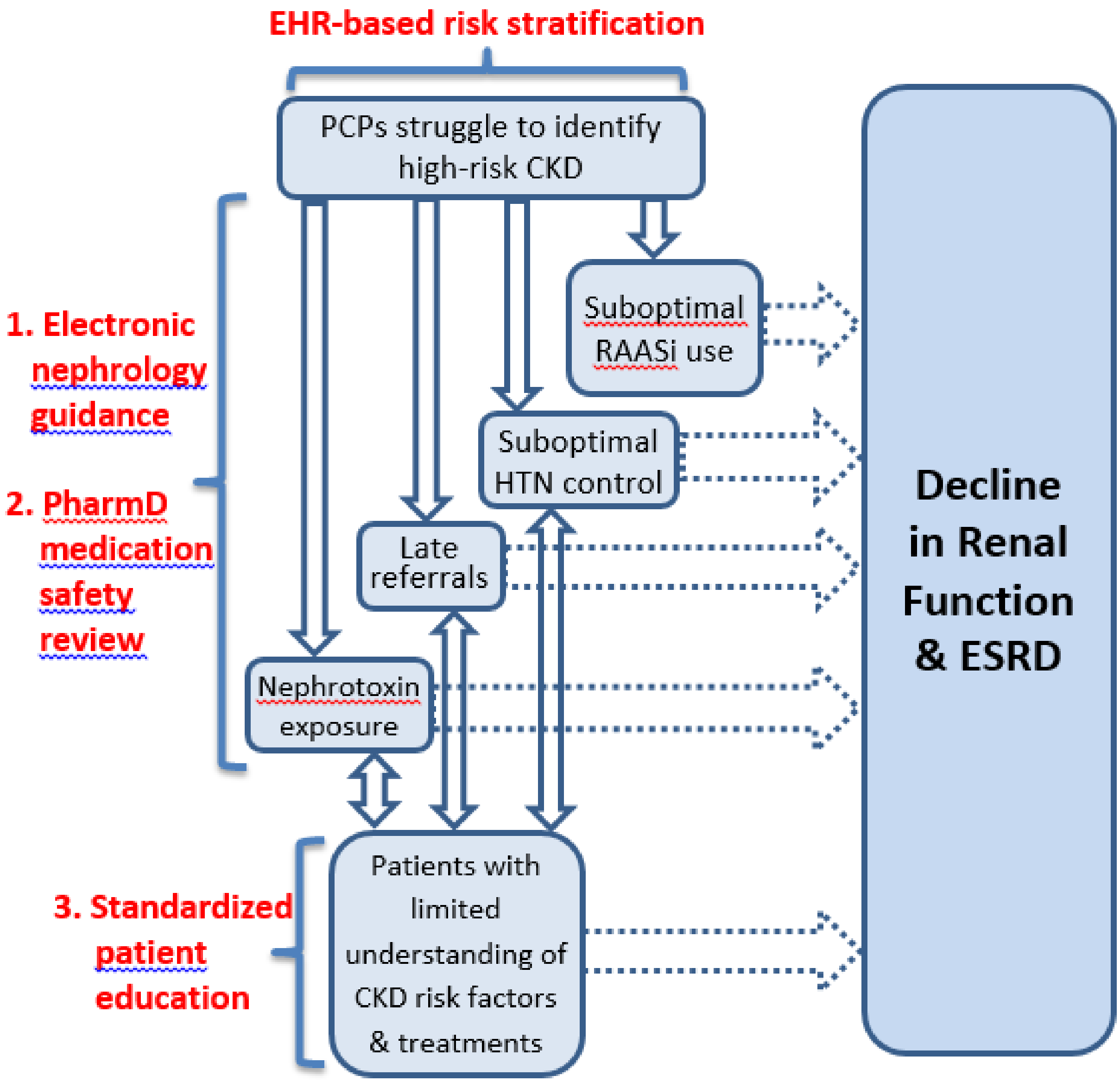

Figure 2. PHM to improve CKD care

III. Study Objectives

The overarching aim of the Kidney Coordinated HeAlth Management Partnership (Kidney CHAMP) trial is to test the effectiveness of a multifaceted EHR-based PHM intervention to improve evidence-based CKD care in high-risk patients in a highly pragmatic, cluster randomized trial (CRT). By combining timely nephrology guidance, pharmacist-led medication management services, and CKD patient education, our intervention will improve CKD risk stratification, resource allocation, adoption of evidence-based interventions, and medication safety and efficacy, while minimizing the PCP and patient burden

Aim 1: To perform a 42-month pragmatic, cluster RCT comparing the effect of an EHR-based PHM intervention versus usual care on key processes of care in 1,700 high-risk CKD patients.
Aim 1a: To examine the effect of the intervention on systolic blood pressure (SBP) in HTN patients.
Aim 1b: To examine the effect of the intervention on RAASi use in albuminuric patients.
Aim 1c: To examine the effect of the intervention on exposures to potentially unsafe medications.

Aim 2: To test the clinical effectiveness of a multifaceted PHM intervention in reducing kidney disease progression in 1,700 high-risk CKD patients enrolled in a 42-month pragmatic, cluster RCT.

IV. **Study Design**

<u>Study design</u>. A 42-month cluster RCT with randomization occurring at the practice level (to minimize contamination) and stratified by number of high-risk CKD patients in the practice. All 90 practices will be randomized. Patient enrollment will continue for 18 months, with a minimum of 24 months of follow-up (**Fig 3**).

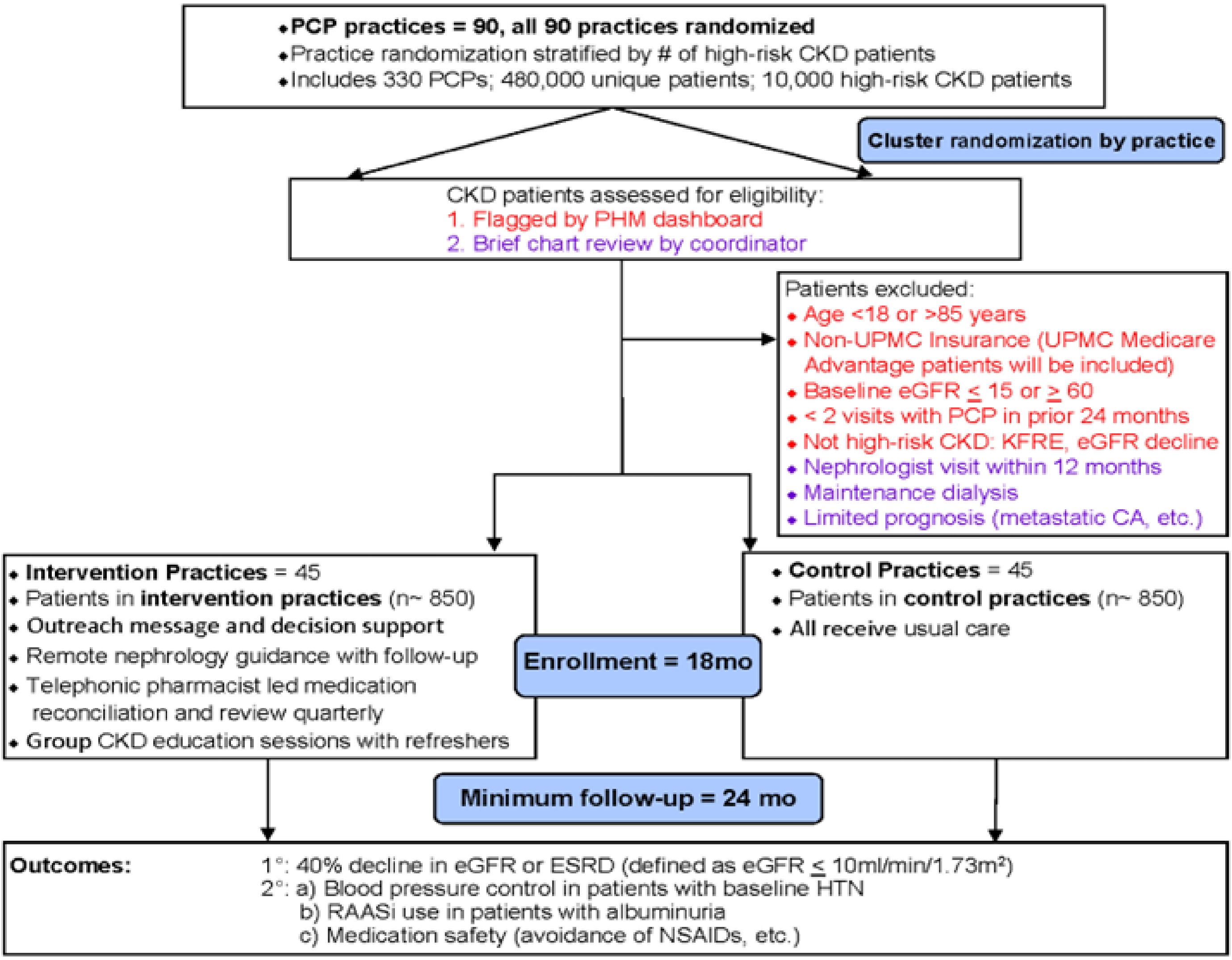

Figure 3. Flow diagram of cluster RCT of EHR-based PHM.
PCP primary care provider, PHM population health management, KFRE kidney failure risk equation, CA cancer

Population and Setting. The study leverages a PCP network that includes 90 practices; 330 PCPs; and over 480,000 patients to conduct a cluster RCT testing the effectiveness of an EHR-based CKD PHM intervention. In this pragmatic study, practices will be randomized to the intervention. Secondarily, their high- risk CKD patients who are not seeing a nephrologist will be enrolled.

Eligibility. Inclusion criteria are (**Figure 3**): a) age ≥ 18 and ≤85, b) most recent eGFR < 60 ml/min/yr,c) UPMC health plan insurance, d) established care with UPMC PCP, e) high-risk CKD (see **Table 1**). Exclusion criteria are: a) history of kidney transplant, b) receiving maintenance dialysis, c) recent (within 12 months) outpatient nephrology visit, d) baseline eGFR ≤ 15ml/min, or e) expected survival ≤ 6 months/hospice.We will use the validated 4- variable KFRE[1,35,83] to estimate 5-year ESRD risk. We will supplement these criteria by including other patients who are high risk for poor outcomes (**Table 1**).[37,74]

Table 1: High risk CKD
eGFR 15-29ml/min OR
5 year risk of ESRD ≥ 4% determined using the validated 4-variable kidney failure risk equation[35,83] (urine dipstick substituted for ACR when necessary) OR
Rapid decline in eGFR operationalized as annualized eGFR decline >=5ml/min/yr[74]*
*determined from 2 eGFR values at least 12 months apart

PCP Recruitment and Engagement.

Before implementation at each site, we will provide a remote/on-site presentation during a scheduled practice meeting. Communication with lead physicians will continue during the study at least biannually by teleconference or on-site lunches to discuss feedback and concerns and document comments on a standardized feedback form. Study/Site PIs will also meet with practice physicians as needed to address concerns. We will anonymously survey intervention arm PCPs *every 6 months* to assess their experience with the intervention bundle. This will include: ease of use, questions communicated by patients, effects on patient medication use and adherence, utility of the recommendations, and temporal burden of the intervention. *Minor adjustments to PHM intervention workflow will be made as needed to ensure continued workflow optimization.*

Randomization. The unit of randomization will be the practice (**Fig. 3**), stratified by estimated number of eligible CKD patients within the practice (small [<15 patients], medium/large [≥15 patients]). Randomization will use a computer generated random number sequence with random block sizes of 4 and 6.

CKD Registry and PHM Dashboard. Our CKD registry includes all outpatients with a recent eGFR < 60 ml/min followed by a UPMC PCP. The CKD registry identifies patients with CKD, phenotypically characterizes them, and stores information on labs, medications of interest, upcoming PCP appointments, and information about CKD outreach (including dates of electronic outreach, medication reviews, patient education, and pending labs/studies). The registry is updated automatically with activity in the patient chart.

Dashboards are medical informatics data representations that can be employed when decisions need to be made about a population of patients. These tools display groups of patients based on clinical characteristics and allow providers to stratify, filter, and sort by relevant variables. Thus, subgroups of patients can be rapidly identified and targeted for more intensive therapy. Dashboards can improve evidence-based care; however, they have been slow to enter clinical

practice because of the sophisticated underlying data requirements.[84,93]

Our CKD PHM dashboard (**Figure 4**) includes population-based reports built off of the registry (e.g., high-risk patients with PCP appointment in October), as well as graphical and tabular displays of key metrics (e.g.,identified subpopulation with albuminuria who are not on RAASi). Population reports accessible through the dashboard will allow the study coordinator to sort patients based on phenotypic data and upcoming PCP appointments, and include filters such as CKD stage, RAASi use, and PCP group. Longitudinal tracking and notation functions interface with the dashboard and allow documentation of study inclusion, and dates of scheduled/completed aspects of the intervention. Dashboard reports will allow the coordinator to rapidly flag patients for urgent concerns, send routine reminders to PCPs to implement recommended interventions, ensure follow-up on pending components of the intervention, and actively monitor follow-up status. Dashboard reports will be actionable and will allow the manager to view more detailed information in a viewing pane without leaving the report. The reports allow the coordinator to jump directly into activities to communicate with the patient, the patient's PCP, and the respective nephrologist or pharmacist.

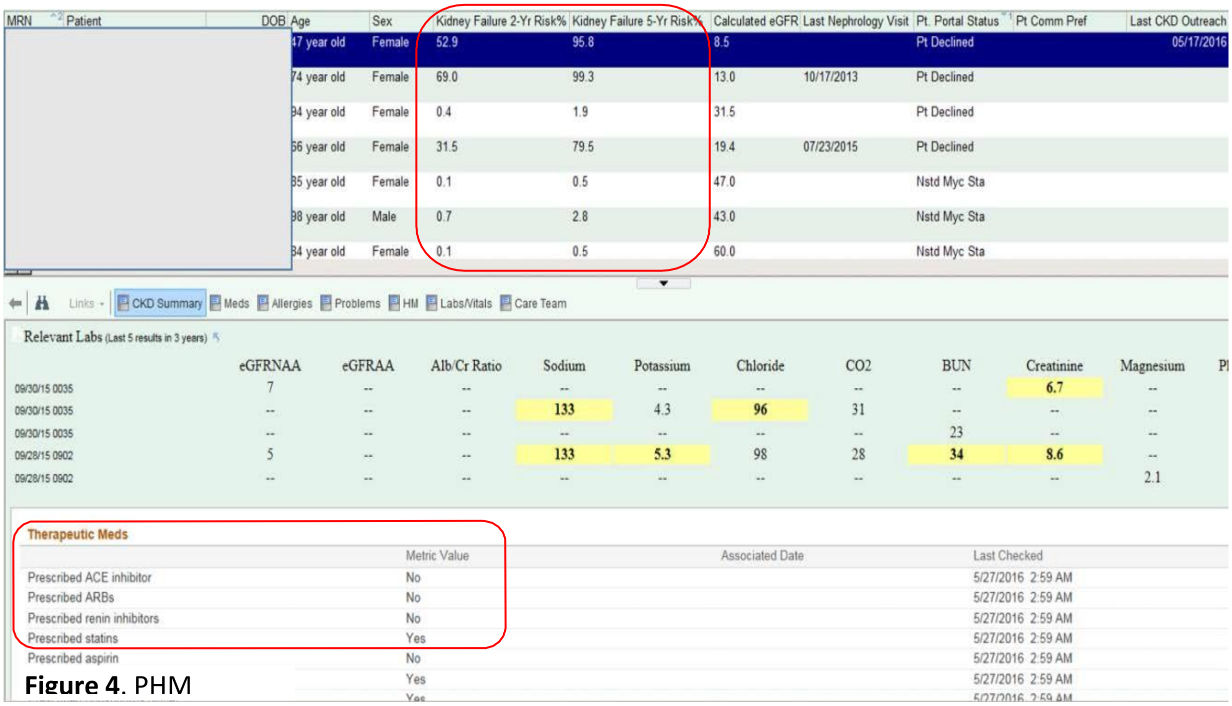

Figure 4. PHM

<u>Patient screening and enrollment</u>. Each month, the coordinator will use the CKD PHM tool to review high-risk patients with an upcoming (within 1 month) appointment with their PCP to determine eligibility. Possible decisions will include actively enroll, permanently exclude, or not presently eligible but may rescreen if deemed high-risk at next PCP appointment. Once delineated, the patient's status is noted in the EHR with a modifiable study flag not visible to providers, but that is continually tracked, updated, and ascertained through the PHM dashboard.

For intervention patients, the coordinator will send a scripted EHR message with associated decision support to the PCP about 1-3 weeks prior to their scheduled PCP appointment. When the PCP accepts the decision support recommendations for an electronic nephrology consultation, a medication reconciliation and safety review, and standardized patient

education, the orders will be placed . *Patients enrolled to practices randomized to usual care will receive care as they currently do*.

A study coordinator is needed to screen patients for eligibility to ensure identical enrollment criteria are used in the intervention and usual care arms. *Because coordinators will review recent patient documentation for potential enrollees in the intervention arm, they will note if a patient has a terminal condition, etc.* However, this information may only be recorded in the text portion of documents. Hence, we will use a standardized form to screen patients in both arms to ensure comparable enrollment. To minimize potential bias from the nurse coordinator's non-blinded status, a local PI blinded to PCP group assignment will review randomly selected screened patients on a weekly basis to ensure accurate implementation of the eligibility criteria. Potential discrepancies will be resolved by consensus and further training as necessary.

<u>Intervention</u>. Approximately 1-3 weeks prior to an appointment, PCPs of enrolled patients will receive an EHR message informing them of the patient's high-risk CKD status. Linked with the notification is a decision support alert (**Figure 5**) that asks the provider to a) order a CKD care bundle that includes an electronic nephrology consultation, pharmacist led medication reconciliation and safety review, and a CKD education session, or b) order a traditional nephrology consult, or c) provide a reason why neither choice is warranted. Notification messages and PCPs' responses will be documented in the EHR.

Figure 5. EHR message linked to decision support alert

Call Documentation

Jhamb, Manisha, MD at 1/11/2016 9:32 AM

Status: Signed Editor: Jhamb, Manisha, MD (Physician)

Sent on behalf of Dr. Francis Solano (President, CMI-UPMC) and Dr. Thomas Kleyman (Division Chief, Renal-Electrolyte Division, University of Pittsburgh)

CMI has partnered with our nephrology division to improve the care of our patients with CKD. Your patient has been identified as high risk to progress to ESRD and may be in need of nephrology input.

Our program will allow you and your patient to receive timely input to help improve the life of kidneys and to make sure the complications of renal disease are being monitored and addressed, i.e., blood pressure control, need for fistula, etc.

Your patient will be seeing you in the next few days. There are 3 options we offer you:

1. Allow us to do an eConsult prior to the visit. This is a new service which we are piloting on a limited basis where a nephrologist will review the chart and make recommendations to you. This may be particularly useful if you are not sure that face-to-face consultation is needed (currently no charge to the patient).

2. Refer to a nephrologist when you see the patient at her upcoming office visit.

3. Choose "No Consult" option, if you do not think any of the above actions are warranted. Please also provide us a reason for this choice.

Please go to the BestPractice section on the Visit Navigator to record your response.
We look forward to helping you care for this patient.

If the care bundle is ordered, a nephrologist will review the chart and provide specific prior to the appointment. In addition, a telephonic appointment with a pharmacist will be made and s/he will contact the patient and review their medication list and provide safety recommendations to the PCP through the EHR.

During the patient visit, a real time decision support alert will remind the PCP to review the nephrology e- consult and pharmacist recommendations, to inform the patient about their CKD status, and to refer the patient for complimentary CKD education. Following the visit, the patient will have their CKD education sessions scheduled. Subsequent nephrology electronic follow-ups will be scheduled according to the patient's clinical needs (generally every 3-6 months) and coordinated through the PHM dashboard. The intervention has been refined to facilitate care enhancements, preserve workflow, and minimize PCP and patient burden.

We have also taken steps to enhance PCP acceptance of the intervention: a) high-level support from UPMC Health Plan, PCP network (Community Medicine Incorporated, including 90 practices and 330 PCPs), and the Nephrology Division; b) meeting with lead physicians and refining the intervention to harmonize with workflow; c) documenting PCP responses in the EHR; d) ongoing communication with PCP groups and leadership.

Electronic nephrology consult. Orders for e-consults will be received in an electronic basket monitored by the study coordinator. The consults will be routed to 1 of 12 board certified nephrology clinicians, who will undergo training to standardize consult focus and communication. The notes will adopt a "Situation, Background, Assessment, Recommendation"[94-97] template to ensure clear, concise communication. The note will be completed at least several days prior to the patient's appointment and routed to the PCP's message basket and documented in the chart to allow time for review and clarification. The initial consult will focus primarily on HTN control , proteinuria assessment and suppression, RAASi use, and medication safety. The note will include a bolded list of recommendations with orders placed in a pended status (i.e., entered, but awaiting acceptance from the PCP). To enhance communication and care coordination, the e- consultant will ensure CKD is added to the problem list.[98] In addition, the problem list will convey that the patient is followed by the e-consult team, reachable via listed message baskets and phone numbers. The nephrologist will include an order for remote electronic follow-up, which will be captured in the PHM dashboard. Electronic messages will be sent to the nephrologist to perform the follow-up approximately 3 weeks before the recommended interval. When necessary, traditional office evaluations will be suggested.

Medication reconciliation and safety review. Prior to their upcoming PCP appointment, patients will be contacted to schedule a remote medication review with a study pharmacist (PharmD) who has expertise in medication therapy management.[43] Appointment availability will include evenings to maximize patient convenience. Patients will be asked to have their medications available for the review. Prior to and during the review, the PharmD will gather clinical data from the EHR's active and recently discontinued medications. S/he will also note the patient's most recent medication dispensing record for each medication (usually documented in the EHR with electronic scripts). The study pharmacist will assess the patient's self- reported medication regimen, administration routine, adherence, and will reconcile this with the medication information contained in the EHR. S/he will assess over the counter (OTC) medications and herbal products, will deliver guidance on OTC medications to avoid, and will screen for possible adverse effects of all medications. Thomas D. Nolin, Associate Professor, PharmD, PhD (**Co-I**), will guide the medication review, perform intermittent audits to ensure intervention fidelity, and direct additional staff training if necessary.[91]

The pharmacist will document their findings in the EHR using a standard medication reconciliation and review template. The note will be sent to the PCP's EHR inbox. Specific, concise recommendations and reasoning will be listed at the top of the note. Thereafter, pharmacist medication reviews will be scheduled quarterly.

Standardized patient education. Once an order for CKD education is placed, the research team will schedule individual or group education session. Caregivers will be encouraged to attend. Study nurse educators will deliver the CKD education. New nurse educators will undergo an intensive 3- to 6-month training period under the guidance of the PIs and existing UPMC CKD nurse educators and dietitians. Simulated and authentic patient education sessions will be observed to judge readiness.

Print and video based education material from the National Kidney Disease Education Program (NKDEP) and the National Kidney Foundation that reviews the role of the kidneys, CKD risk factors, dietary guidelines, pharmacotherapy, medication adherence and safety, frequently asked questions, and dialysis modalities will be used. The nurse will document

the session in the EHR using a brief templated education note (that is captured by the PHM dashboard), and route it to the PCP and nephrologist. After the initial sessions, annual refresher sessions will be scheduled.

Multi-disciplinary Case Discussions

Prior to providing recommendations to PCPs, every patient's management will be discussed in case conference calls (2-4 times per week) attended by APPs, nephrologists, and pharmacists, to arrive at consensual individualized recommendations for patients.

Intervention Fidelity

All nephrologists, nurse educators, and PharmDs delivering a component of the intervention will undergo standardized training until they achieve consistent and acceptable performance. This will include review of concise educational materials, observation and apprenticeship with existing providers at the local site, the use of checklists operationalizing key aspects of each intervention, the use of SBAR (situation, background, assessment, recommendation) EHR templates,[94-97] and direct observation during role played and actual interventions. After initial implementation, the study/site PIs will randomly audit 5-10% of e- consults every 3 months. Dr. Nolin will randomly audit 5-10% of pharmacy communications every 3 months. Providers will receive targeted feedback based on findings. Providers will also continue to use checklists to document completion of key aspects of the intervention as well as deviations throughout the study. Refreshers will occur every 6-12 months and remediation will occur as dictated by observed performance (i.e., <80% fidelity with items on checklist).

V. Data collection and Outcomes

Routinely collected EHR and administrative data will be abstracted for outcomes assessment as shown in Table 2. PCP practice level data will be obtained from public records.

Table 2: Key variables and covariates for usual care and intervention patients

Variables	Variable descriptions	Data source details
Renal function and rate of change in renal function	• Baseline serum creatinine – most recent creatinine from date of study enrollment visit with PCP up to 365 days prior to the visit.<br>• Baseline eGFR – Calculated using CKD-EPI.[99]<br>• Baseline rates of change in eGFR - determined from baseline eGFR and prior eGFRs between 365 to 730 days before the baseline value.	EHR (restricted to outpatient labs)
Socio-demographics	• Age, gender, race, ethnicity, marital status, insurance, and zip code for linkage with neighborhood median household income.[39]<br>• Baseline values defined on the date of baseline visit	EHR
Comorbid conditions	• DM, HTN, hyperlipidemia, CAD, cerebrovascular disease, peripheral vascular disease, CHF, arrhythmia, gout, chronic lung disease, chronic liver disease, mood disorder, and malignancy.<br>• Baseline values defined on the date of baseline visit and using a 24 month "look back" period.	Phenotypes using administrative & clinical codes, meds, & lab values validated in local EHR.[12,38,39]
Blood Pressure	• Baseline BP - mean outpatient BP from the date of the baseline study visit with the PCP until 180 days prior to the baseline visit.<br>• Follow-up BP – all outpatient BPs after patient enrollment	Office visit vital signs recorded in the EHR
Medication use	• RAASi, NSAID and other medications deemed a potential safety concern (e.g., allopurinol, gemfibrozil, glyburide, metformin, etc.)<br>• Medication related problems and drug record discrepancies	EHR medication list; medication review (intervention patients)
Laboratory values	• Common laboratory tests (e.g., *K+*, cholesterol, etc.)<br>• Baseline values - determined using the most recent value from baseline visit up to 365 days prior to the visit.	EHR (restricted to outpatient labs)
Urine albuminuria	• Quantitative urine albuminuria - most recent ACR from the baseline visit up to 365 days prior to the visit.<br>• Urine dipstick albuminuria - median of outpatient values available from the date of baseline visit up to 365 days prior to the visit.[100]	Outpatient lab values from EHR

Primary Outcome

A ≥40% decline in eGFR or ESRD.[92] eGFR decline will be adjudicated based on the baseline creatinine and eGFR determined from the CKD-EPI equation and measured routinely in clinical practice.[99] ESRD will be defined as an eGFR ≤ 10ml/min to account for patients with markedly reduced baseline eGFR values (i.e., 16-20ml/min).

The 40% decline surrogate outcome may increase power and precision by capturing additional events while maintaining a similar risk of type I error[92] compared to the standard doubling of serum creatinine outcome. To limit surveillance bias, we will use 6-month ascertainment windows and average all values within each window. Our data indicate 75% of high-risk patients have an outpatient eGFR value every 6-months. Additional analyses will compare changes in eGFR slope over time (using splines to account for non-linearity.

Secondary outcomes - Process of care outcomes

1. HTN control. Outpatient, sitting BP values measured during each outpatient encounter and recorded in the EHR. BP will be treated as a continuous variable. To minimize

ascertainment bias, we will use 6-month ascertainment windows to determine an average BP for each patient for each 6-mo period. Patients lacking an outpatient value will have their last value carried forward

2. Use of RAASi. Will be determined by active use of an ACEi or ARB based on the EHR medication list at each outpatient encounter. Analyses will compare cumulative person-time exposure during the study.

3. Medication safety. We will examine the rates of use of several high-risk medications[21,43,54,61,80,101] that can be associated with adverse outcomes in progressive CKD. Medication exposure will be determined by presence of the specified medication on the patient's EHR medication list at each outpatient encounter. Analyses will compare cumulative person-time exposure during the study.
    a. Use of NSAIDs: use examined for all study patients
    b. Use of glyburide: use examined for all diabetic study patients
    c. Use of metformin: use examined for diabetic study patients with eGFR<30
    d. Use of gemfibrozil: use examined for all study patients with eGFR<30.

Exploratory outcomes. Mortality, hyperkalemia, and health utilization (i.e., costs) including hospitalizations, emergency department visits, and outpatient encounters will be ascertained by a combination of EHR and administrative data from the UPMC health plan. The accessibility of administrative data that captures events outside the health system and supplements the EHR is a unique and complementary resource that will be leveraged in future study analyses.

Adverse events

While it is unlikely that systematically providing evidence-based recommendations to PCPs of high-risk CKD patients by trained nephrologists and pharmacists will worsen overall clinical outcomes, we will monitor safety signals through the EHR, including hyperkalemia, ER visits, hospitalizations and mortality.

VI. **Regulatory and Oversight Considerations**

This study meets the criteria for human subject's research.

We will conduct a cluster RCT of PCP practices, implementing a multifaceted PHM intervention for patients with high-risk CKD. The intervention targets improvements in the delivery of evidence-based care and outcomes. Patients with high-risk CKD will be identified through demographics and laboratory tests that are collected for clinical purposes. The PHM dashboard, which includes estimates from validated risk prediction models and eGFR trajectories, will be used to identify and track enrolled patients. Shortly before their regularly scheduled visit with an enrolled patient, PCPs randomized to the intervention arm will receive a decision support message and subsequent reminders notifying the provider of the patient's high-risk CKD status and recommending the following bundle: 1) an order for electronic nephrology guidance or formal nephrology office consultation if preferred; 2) medication therapy management by a PharmD, specifically including a medication reconciliation and safety review, and 3) CKD patient education. These interventions are all consistent with standard of care practices for patients with high-risk CKD and the PCP will always be permitted to accept or refuse these suggestions according to their clinical judgement. If the provider accepts these suggestions, the individual intervention components will be ordered, scheduled, coordinated, and tracked. If the provider refuses any of the components, they will be able to justify why their reasons for refusal. Refusal responses will be randomly audited to ensure accuracy.

a. Recruitment and Informed Consent:

We will include all primary care providers at UPMC with an active primary care continuity clinic. We will identify these providers through provider rosters given to us by the respective departments and practices. We will maintain contact with each practice through biannual sessions held during their regular practice meetings or lunch meetings held at their offices. We will remind providers of the aims of the study and the intervention. We will also seek regular feedback from providers to identify unforeseen issues that were not encountered during the pilot study and will work collaboratively with them to identify suitable solutions. We will survey providers on their experience with the intervention using both multiple choice and open-ended questions. We will also collect feedback from them using standardized, templated forms during planned meetings. The study's PIs and nurses will meet with the practices as described above.

Per the University of Pittsburgh IRB and UPMC Quality Improvement committee guidance, the study will not require consent from PCP or patient to enroll them in the study. Both PCP and patients will be given information about the study and an opportunity to opt-out. We will continue to meet with PCPs to understand barriers that may arise and to develop solutions that ensure PCP burden is minimized and workflow is preserved.

Notably, all practicing PCPs who partake in this study are licensed providers. They vary in age from approximately 30 years to >60 years. All PCPs from UPMC primary care practices are included in this research; no one is excluded. The PCPs care for approximately 480,000 patients annually, including ~10,000 patients with high-risk CKD. Individual patients seen will vary greatly in age and health status as expected in any large group PCP practice.

The intervention will only target patients who have high-risk CKD using validated risk prediction models. Low- risk patients will be excluded because outcomes occur at a lower rate in this setting. Including these patients would necessitate a larger, longer, and costlier study to ensure adequate power to detect small but meaningful differences. Alternatively,

including these patients using the current study size and follow-up would result in an underpowered trial with significant potential for a type II error. Patients > 85 years of age will be excluded as best practices for CKD treatment are less clear in the very aged. Patients < 18 years will also be excluded (see inclusion of children below). Patients with a history of renal transplant, end-stage renal disease, or already under the care of a nephrologist will be excluded because these patients are generally receiving specialized care in addition to their PCP's care. In addition, patients with very limited prognoses (e.g., metastatic cancer, COPD on continuous oxygen, stage IV heart failure) will be excluded *during the screening process* due to the difficulty of substantially altering their course with CKD treatment enhancements.

Patients that are pregnant or prisoners will be excluded from the study if they are seen by a participating PCP during the study enrollment period. Evidence-based CKD care differs during pregnancy (e.g., contraindication of RAASi, different BP goals) and pregnancy and peripartum course are known to affect eGFR and albuminuria thereby uniquely affecting potential outcomes. In addition, because 2 components of our intervention bundle require scheduling a group CKD education session and a telephonic medication reconciliation and safety review with a PharmD, enrolling incarcerated patients would be impracticable.

a. *Potential Risk:*

PCPs will be at minimal risk with regards to their reputation, finances, legal liability, or position in their department or practice. Their exposure is limited to intermittent outreach from the investigators and EHR communications/reminders regarding their enrolled patients. These messages will recommend evidence-based care (e.g., implementation of RAASi, avoidance of NSAIDs, checking a urinary albumin to creatinine ratio) for a high-risk CKD population. The provider can then choose to enact or ignore the suggestions, and document reasons for refusal. The messages and communication have been designed to activate in a manner that harmonizes with existing PCP workflow. Processing these messages should require far less than 1 minute per patient, exposing the PCPs to a minimal risk of temporal inconvenience. Assuming a uniform distribution of high-risk CKD patients throughout the practices, the average PCP will have ~5 patients included in the study.
However, because patient distribution is not uniform, we estimate a single PCP may have up to ~15 patients in the study. This will still pose a minimal temporal burden over the course of the trial.

All data analysis on provider performance will be reported in the aggregate; hence, performance of an individual PCP will not be identifiable. In the unlikely event of a breach of confidentiality, the physicians will be exposed to minimal risks to their reputation, job security/finances, or legal liability as physician data will be strictly de-identified with a password protected "key" stored separately in a password protected file on a secure university server. Even if the key was stolen (which we deem to be very unlikely to happen), the data regarding physicians would be of limited implication as there are no formal metrics regarding CKD care and we will not gather any high-risk provider information (e.g., dob, SSN, etc). Alternative treatments at this time are to continue usual care (e.g., continuing medical education activities to PCPs) which has proven ineffective in optimizing PCP performance including treatment of CKD.

This study will also subject the enrolled patients of participating providers to minimal risks. At baseline, enrolled patients are at high-risk for poor outcomes including catastrophic outcomes such as ESRD. The predictive models used to make these estimates have been externally validated in multiple populations. When the PCP accepts the recommended intervention bundle the following events will occur. First, electronic nephrology guidance will

be provided, which will give the PCP recommendations on how to improve CKD related care prior to their visit with the patient. This intervention is strictly provider facing (i.e., the patient is not contacted by the nephrologist). The PHM dashboard will be used to track whether recommendations are implemented and to send reminder messages to PCPs unless reasons for non-implementation are provided. Second, the patient will have a telephonic appointment made with a PharmD to review their medications. If an emergent medication hazard is discovered, the PharmD will ask the patient to hold the offending medication(s) and will immediately contact PIs, and the patient's PCP. These emergent events are likely to be quite infrequent. Otherwise, following completion of the medication reconciliation and safety review, the pharmacist's findings will be reported to the PCP so that they may be reviewed with the patient and appropriate changes made (in accordance with the PCP's clinical judgement). Third, the patient will be scheduled for a CKD nurse education session following their appointment with their PCP (thereby allowing the PCP to share the CKD diagnosis with the patient). The nurse education session will provide information on the role of the kidneys, kidney function assessment, strategies to protect kidney function, medication safety, and general information about ESRD treatment options. Given patients' high-risk CKD status, CKD education isconsidered standard of care and patients may opt out of the education session if they find it distressing.

Some of these interventions may pose a small psychological risk to the patient. Patients who were previously unaware, may become aware of their CKD status. However, the patient will have discovered the presence of a serious illness with potentially severe complications and (most importantly) available treatments. Further studies to delineate the exact etiology of the CKD and subsequent treatments can be initiated. The intervention may assist with proper medication dosing, avoidance of nephrotoxic medications, and avoidance of potentially risky procedures (imaging with intravenous contrast or gadolinium containing compounds). The intervention may also help delay the need for dialysis and ensure patients are prepared for dialysis if it becomes necessary. In addition, PCPs can choose to defer discussions and treatment if they feel a patient is unlikely to have high- risk CKD or benefit from any of the aspects of the intervention. The presently available alternative approach is to continue current practice with suboptimal PCP treatment of CKD.

While it is unlikely that systematically providing evidence-based CKD recommendations to PCPs of high risk patients by board certified nephrologists and pharmacists will worsen overall clinical outcomes, we will monitor several safety signals. We will gather these safety data annually through the EHR, minimizing additional patient or study cost burden. Potential adverse events that will be monitored include:

1) Rates of hyperkalemia ($K>5.5$, $K\geq6$)
2) Rates of emergency department visits and hospitalizations
3) Rates of death.

Tests and treatments implemented by PCPs based on nephrologist's or pharmacist's recommendations are likely to be relatively inexpensive and non-invasive and should expose the patient to minimal financial or bodily risk. *The board certified PCP is the final arbiter of medical decisions regarding their patients*.

No patient will be excluded based on gender, race, or ethnicity. However, we are restricting the intervention to UPMC health plan patients (including UPMC Medicare Advantage) for several reasons. First, this allows us to inform PCPs and patients that we have partnered with the patient's insurer in an effort to optimize care, thereby mitigating potential financial concerns. Second, the health plan's support will allow us to supplement EHR data with administrative

data to adjudicate outcomes while minimizing misclassification. Our findings will inform future efforts to extend the intervention while preserving fiscal sustainability.

b. Protections against Risk:
Data will be stored in secured databases (e.g., MS access, REDCap) on secure university servers accessible only through password protected computers in locked rooms. All working datasets will be de-identified limited datasets (i.e., dates of tests, labs will remain). Identifiers will be stored in a separate, password protected file. Access to the PHM dashboard similarly requires 1) a valid password to access the university computer, 2) a valid password to access the EHR, and 3) clearance/privileges to access the PHM dashboard.

The risk of breach of confidentiality is low. Further, all information associated with provider performance or patient information will be de-identified (using limited data sets with dates). Hence, if there is a breach in confidentiality it is unlikely to expose the providers or patients to any significant harm or discomfort. Given the lack of published literature on the usefulness or futility of PHM in CKD, breach of confidentiality regarding provider participation or randomization assignment is also very unlikely to result in any foreseeable harm or discomfort. Patient information will be de-identified and high-risk variables (e.g., DOB, etc) removed from all working datasets to minimize the risk of breach of confidentiality. The minimum data necessary for the study will be accessed. In addition, all researchers involved are clinically competent and certified in HIPAA compliance. All stored electronic data will be kept on University secure computers and secure servers in locked departmental offices. The corresponding electronic databases are password protected. All paper records associated with the study will be stored in a locked file cabinet in a locked room. These measures are likely to be effective.

To minimize the risks of the PHM intervention (e.g., possible psychological distress), we are limiting our intervention to patients with high-risk CKD based on clinically validated risk prediction models. This model will limit the targeted population thereby restricting those subjected to the above-mentioned risks. Additionally, all communications are targeted to licensed providers who will exercise their clinical judgment and can ignore recommendations they feel would subject the patient to an undue burden. In this manner, the intervention is non-invasive and nonbinding (i.e., it is always left to the provider's discretion whether to follow suggestions). Together, these are likely to successfully limit unnecessary interventions and the placement of undue psychological or financial risks on patients.

<u>Data Safety and Monitoring Plan</u>.
A data and safety monitoring plan (DSMP) will be implemented by Drs. Jhamb and Abdel-Kader, and members of the research team, to ensure that there are no changes in the risk/benefit ratio during the course of the study and that confidentiality of research data is maintained. Investigators and study personnel will meet monthly to discuss the study (e.g. study goals and modifications of those goals; subject recruitment and retention; progress in data coding and analysis; documentation, identification of adverse events or research subject complaints; violations of confidentiality) and address any issues or concerns at that time. Minutes will be kept for these meetings and will be maintained in the study regulatory binder. The status of recruitment and data collection will be discussed and addressed among the attendees with confirmation that proper protocol has been followed. Technical problems if any will be discussed and plans developed to address them. Any instances of serious adverse events will be reported immediately to the University of Pittsburgh IRB using standard forms and/or procedures that have been established by the IRB.

The yearly IRB renewal for this study will include a summary report of the DSMP findings from the prior year.

Inclusion of Women and Minorities:

Given the study design of randomizing practices, the subject selection criteria are all PCP practices previously specified. Secondarily, the patients with high-risk CKD who are seen by a participating PCP will be included. We are unable to control the gender, race, or ethnicity of the PCPs or of their patients. However, we will include all PCPs regardless of gender, race, or ethnicity. We will also include all of their eligible patients between the ages of 18 and 85 who have high-risk CKD. No patient will be excluded based on their gender or race or ethnicity. Indeed, because patients with high-risk CKD are often of minority race or ethnicity, we expect higher proportions of these groups than the general population. However, there will not be any proposed outreach program for recruiting members of a specific gender or racial/ethnic group as subjects. We do not suspect that one gender or racial group will be excluded or underrepresented given the baseline patient demographics of the PCP practices (55% women, 15% African-Americans) and data from the USRDS annual report revealing that nearly 30% of incident dialysis patients are African-American and that women have a greater prevalence of CKD stages 3-5. While we acknowledge that the local Hispanic/Latino-American population is relatively small compared to the national average, we will attempt to include every patient deemed to have high-risk CKD seen by a participating PCP. The racial and ethnic diversity of patients included in the study will be entirely based on the diversity of the local PCP practices and is outside our control. We will not exclude any patient based on their gender, race, or ethnicity.

Inclusion of Children

Participants in this study are enrolled at two levels. First, we are directly enrolling PCPs. None of the PCPs are children and hence no children will be recruited at this stage. However, patients with high-risk CKD are secondarily included in the study when they are seen by a participating PCP. The participating PCPs generally see patients >18 years old (some family practice physicians see both children and adults). However, we will only be targeting patients ≥ 18 years. Hence, children will not be included. This is justified for several reasons:

1) the prevalence of CKD in this age range is low and there will be few patients who meet these criteria, 2) CKD in the adult population has different etiologies, natural history, and treatments, 3) well validated risk prediction models to determine high-risk status are not available to our knowledge. All of these reasons make including children impracticable in this study.

References

1. Tangri N, Grams ME, Levey AS, et al. Multinational assessment of accuracy of equations for predicting risk of kidney failure: A meta-analysis. *JAMA.* 2016;315(2).
2. Lipworth L, Abdel-Kader K, Morse J, et al. High prevalence of non-steroidal anti-inflammatory drug use among acute kidney injury survivors in the southern community cohort study. *BMC Nephrol.* Nov 24 2016;17(1):189.
3. Siew ED, Parr SK, Abdel-Kader K, et al. Predictors of Recurrent AKI. *J Am Soc Nephrol.* Apr 2016;27(4):1190-1200.
4. Matheny ME, Peterson JF, Eden SK, et al. Laboratory test surveillance following acute kidney injury. *PloS one.* 2014;9(8):e103746.
5. Loudon K, Treweek S, Sullivan F, Donnan P, Thorpe KE, Zwarenstein M. The PRECIS-2 tool: designing trials that are fit for purpose. *BMJ.* May 8 2015;350:h2147.
6. Coresh J, Selvin E, Stevens LA, et al. Prevalence of chronic kidney disease in the United States. *JAMA.* Nov 7 2007;298(17):2038-2047.
7. Hoerger TJ, Simpson SA, Yarnoff BO, et al. The future burden of CKD in the United States: a simulation model for the CDC CKD Initiative. *Am J Kidney Dis.* Mar 2015;65(3):403-411.
8. Honeycutt AA, Segel JE, Zhuo X, Hoerger TJ, Imai K, Williams D. Medical costs of CKD in the Medicare population. *J Am Soc Nephrol.* Sep 2013;24(9):1478-1483.
9. Go AS, Chertow GM, Fan D, McCulloch CE, Hsu CY. Chronic kidney disease and the risks of death, cardiovascular events, and hospitalization. *N Engl J Med.* Sep 23 2004;351(13):1296-1305.
10. Samal L, Wright A, Waikar S, Linder J. Nephrology co-management versus primary care solo management for early chronic kidney disease: a retrospective cross-sectional analysis. *BMC Nephrology.* 2015;16(1):162.
11. Richards N, Harris K, Whitfield M, et al. The impact of population-based identification of chronic kidney disease using estimated glomerular filtration rate (eGFR) reporting. *Nephrol Dial Transplant.* Feb 2008;23(2):556-561.
12. Abdel-Kader K, Fischer GS, Johnston JR, Gu C, Moore CG, Unruh ML. Characterizing pre-dialysis care in the era of eGFR reporting: a cohort study. *BMC Nephrol.* 2011;12:12.
13. Lee BJ, Forbes K. The role of specialists in managing the health of populations with chronic illness: the example of chronic kidney disease. *BMJ.* 2009;339:b2395.
14. Abdel-Kader K, Greer RC, Boulware LE, Unruh ML. Primary care physicians' familiarity, beliefs, and perceived barriers to practice guidelines in non-diabetic CKD: a survey study. *BMC Nephrol.* 2014;15:64.
15. Boulware LE, Troll MU, Jaar BG, Myers DI, Powe NR. Identification and referral of patients with progressive CKD: a national study. *Am J Kidney Dis.* Aug 2006;48(2):192-204.
16. Greer RC, Cooper LA, Crews DC, Powe NR, Boulware LE. Quality of patient-physician discussions about CKD in primary care: a cross-sectional study. *Am J Kidney Dis.* Apr 2011;57(4):583-591.
17. Greer RC, Crews DC, Boulware LE. Challenges perceived by primary care providers to educating patients about chronic kidney disease. *Journal of renal care.* Dec 2012;38(4):174-181.
18. Vest BM, York TR, Sand J, Fox CH, Kahn LS. Chronic Kidney Disease Guideline Implementation in Primary Care: A Qualitative Report from the TRANSLATE CKD Study. *J Am Board Fam Med.*

Sep-Oct 2015;28(5):624-631.
19. United States Renal Data System. 2015 USRDS annual data report: Epidemiology of kidney disease in the United States. National Institutes of Health, National Institute of Diabetes and Digestive and Kidney Diseases, Bethesda, MD, 2015. 2015.
20. Chan MR, Dall AT, Fletcher KE, Lu N, Trivedi H. Outcomes in patients with chronic kidney disease referred late to nephrologists: a meta-analysis. *Am J Med.* Dec 2007;120(12):1063-1070.
21. Chang F, O'Hare AM, Miao Y, Steinman MA. Use of Renally Inappropriate Medications in Older Veterans: A National Study. *Journal of the American Geriatrics Society.* 2015:n/a-n/a.
22. Fox CH, Swanson A, Kahn LS, Glaser K, Murray BM. Improving chronic kidney disease care in primary care practices: an upstate New York practice-based research network (UNYNET) study. *J Am Board Fam Med.* Nov- Dec 2008;21(6):522-530.
23. Philipneri MD, Rocca Rey LA, Schnitzler MA, et al. Delivery patterns of recommended chronic kidney disease care in clinical practice: administrative claims-based analysis and systematic literature review. *Clinical and experimental nephrology.* Feb 2008;12(1):41-52.
24. Plantinga LC, Miller ER, Stevens LA, et al. Blood Pressure Control Among Persons Without and With Chronic Kidney Disease: US Trends and Risk Factors 1999–2006. *Hypertension.* July 1, 2009 2009;54(1):47-56.

25. Smart NA, Dieberg G, Ladhani M, Titus T. Early referral to specialist nephrology services for preventing the progression to end-stage kidney disease. *The Cochrane database of systematic reviews.* 2014;6:CD007333.

26. Smart NA, Titus TT. Outcomes of early versus late nephrology referral in chronic kidney disease: a systematic review. *Am J Med.* Nov 2011;124(11):1073-1080 e1072.

27. Wyatt C, Konduri V, Eng J, Rohatgi R. Reporting of estimated GFR in the primary care clinic. *Am J Kidney Dis.* May 2007;49(5):634-641.

28. Brenner BM, Cooper ME, de Zeeuw D, et al. Effects of Losartan on Renal and Cardiovascular Outcomes in Patients with Type 2 Diabetes and Nephropathy. *New England Journal of Medicine.* 2001;345(12):861-869.

29. Lewis EJ, Hunsicker LG, Clarke WR, et al. Renoprotective Effect of the Angiotensin-Receptor Antagonist Irbesartan in Patients with Nephropathy Due to Type 2 Diabetes. *New England Journal of Medicine.* 2001;345(12):851-860.

30. Fishbane S, Hazzan AD, Halinski C, Mathew AT. Challenges and opportunities in late-stage chronic kidney disease. *Clinical Kidney Journal.* December 2, 2014 2014.

31. Drawz PE, Archdeacon P, McDonald CJ, et al. CKD as a Model for Improving Chronic Disease Care through Electronic Health Records. *Clin J Am Soc Nephrol.* Aug 7 2015;10(8):1488-1499.

32. Narva AS. Decision Support and CKD: Not There Yet. *Clinical Journal of the American Society of Nephrology.* April 1, 2012 2012;7(4):525-526.

33. Narva AS, Norton JM, Boulware LE. Educating Patients about CKD: The Path to Self-Management and Patient- Centered Care. *Clin J Am Soc Nephrol.* Nov 4 2015.

34. Tuot DS, Diamantidis CJ, Corbett CF, et al. The last mile: translational research to improve CKD outcomes. *Clin J Am Soc Nephrol.* Oct 7 2014;9(10):1802-1805.

35. Tangri N, Stevens LA, Griffith J, et al. A predictive model for progression of chronic kidney disease to kidney failure. *JAMA.* Apr 20 2011;305(15):1553-1559.

36. Tangri N, Kitsios GD, Inker LA, et al. Risk prediction models for patients with chronic kidney disease: a systematic review. *Ann Intern Med.* Apr 16 2013;158(8):596-603.

37. Turin TC, Coresh J, Tonelli M, et al. Short-term change in kidney function and risk of end-stage renal disease. *Nephrology Dialysis Transplantation.* October 1, 2012 2012;27(10):3835-3843.

38. Abdel-Kader K, Fischer GS, Li J, Moore CG, Hess R, Unruh ML. Automated clinical reminders for primary care providers in the care of CKD: a small cluster-randomized controlled trial. *Am J Kidney Dis.* Dec 2011;58(6):894- 902.

39. Jhamb M, Cavanaugh KL, Bian A, et al. Disparities in Electronic Health Record Patient Portal Use in Nephrology Clinics. *Clin J Am Soc Nephrol.* Nov 06 2015;10(11):2013-2022.

40. Wright JT, Jr., Bakris G, Greene T, et al. Effect of blood pressure lowering and antihypertensive drug class on progression of hypertensive kidney disease: results from the AASK trial.[see comment]. *Jama.* 2002;288(19):2421-2431.

41. Appel LJ, Wright JT, Greene T, et al. Intensive Blood-Pressure Control in Hypertensive Chronic Kidney Disease. *New England Journal of Medicine.* 2010;363(10):918-929.

42. Group SR, Wright JT, Jr., Williamson JD, et al. A Randomized Trial of Intensive versus Standard Blood-Pressure Control. *N Engl J Med.* Nov 26 2015;373(22):2103-2116.

43. St Peter WL, Wazny LD, Patel UD. New Models of CKD Care Including Pharmacists: Improving Medication Reconciliation and Medication Management. *Current opinion in nephrology and hypertension.* 2013;22(6):656- 662.

44. Beddhu S, Rocco MV, Toto R, et al. Effects of Intensive Systolic Blood Pressure Control on Kidney and Cardiovascular Outcomes in Persons Without Kidney Disease: A Secondary Analysis of a Randomized Trial. *Ann Intern Med.* Sep 19 2017;167(6):375-383.

45. Abdel-Kader K. The Times, They Are A-Changin: Innovations in Health Care Delivery To Reduce CKD Progression. *Clin J Am Soc Nephrol.* Sep 7 2017;12(9):1375-1376.

46. Krist AH, Beasley JW, Crosson JC, et al. Electronic health record functionality needed to better support primary care. *Journal of the American Medical Informatics Association.* 2014-09-01 00:00:00 2014;21(5):764-771.

47. Institute of Medicine (U.S.) Committee on Quality of Health Care in America. *Crossing the quality chasm : a new health system for the 21st century.* Washington, D.C.: National Academy Press; 2001.

48. Kung HC, Hoyert DL, Xu J, Murphy SL. Deaths: final data for 2005. *Natl Vital Stat Rep.* Apr 24 2008;56(10):1-120.

49. Collins AJ, Foley R, Herzog C, et al. Excerpts from the United States Renal Data System 2007 annual data report. *Am J Kidney Dis.* Jan 2008;51(1 Suppl 1):S1-320.

50. Jolly SE, Navaneethan SD, Schold JD, et al. CKD in an Electronic Health Record Problem List: Quality of Care, ESRD, and Mortality. *American journal of nephrology.* 04/01 2014;39(4):288-296.

51. Rutkowski M, Mann W, Derose S, et al. Implementing KDOQI CKD Definition and Staging Guidelines in Southern California Kaiser Permanente. *American Journal of Kidney Diseases.*53(3):S86-S99.

52. Shahinian VB, Saran R. The Role of Primary Care in the Management of the Chronic Kidney Disease Population. *Advances in Chronic Kidney Disease.* 5// 2010;17(3):246-253.

53. Agrawal V, Ghosh AK, Barnes MA, McCullough PA. Awareness and knowledge of clinical practice guidelines for CKD among internal medicine residents: a national online survey. *Am J Kidney Dis.* Dec 2008;52(6):1061-1069.

54. Allen AS, Forman JP, Orav EJ, Bates DW, Denker BM, Sequist TD. Primary care management of chronic kidney disease. *Journal of general internal medicine.* Apr 2011;26(4):386-392.

55. Plantinga LC, Miller ER, 3rd, Stevens LA, et al. Blood pressure control among persons without and with chronic kidney disease: US trends and risk factors 1999-2006. *Hypertension.* Jul 2009;54(1):47-56.

56. Sakhuja A, Textor SC, Taler SJ. Uncontrolled hypertension by the 2014 evidence-based guideline: results from NHANES 2011–2012. *Journal of Hypertension.* 2015;33(3):644-652.

57. Curtis BM, Barrett BJ, Djurdjev O, Singer J, Levin A. Evaluation and treatment of CKD patients before and at their first nephrologist encounter in Canada. *Am J Kidney Dis.* Nov 2007;50(5):733-742.

58. Chertow GM, Lee J, Kuperman GJ, et al. Guided medication dosing for inpatients with renal insufficiency. *JAMA.* Dec 12 2001;286(22):2839-2844.

59. Diamantidis CJ, Ginsberg JS, Yoffe M, et al. Remote Usability Testing and Satisfaction with a Mobile Health Medication Inquiry System in CKD. *Clin J Am Soc Nephrol.* Aug 07 2015;10(8):1364-1370.

60. Fink JC, Chertow GM. Medication errors in chronic kidney disease: one piece in the patient safety puzzle. *Kidney Int.* Dec 2009;76(11):1123-1125.

61. Hanlon JT, Wang X, Handler SM, et al. Potentially inappropriate prescribing of primarily renally cleared medications for older veterans affairs nursing home patients. *Journal of the American Medical Directors Association.* Jun 2011;12(5):377-383.

62. Taal MW. Slowing the progression of adult chronic kidney disease: therapeutic advances. *Drugs.* 2004;64(20):2273-2289.

63. Effect of intensive therapy on the development and progression of diabetic nephropathy in the Diabetes Control and Complications Trial. The Diabetes Control and Complications (DCCT) Research Group. *Kidney Int.* Jun 1995;47(6):1703-1720.

64. Brown WW, Peters RM, Ohmit SE, et al. Early detection of kidney disease in community settings: the Kidney Early Evaluation Program (KEEP). *Am J Kidney Dis.* Jul 2003;42(1):22-35.

65. McAlister FA, Stewart S, Ferrua S, McMurray JJJV. Multidisciplinary strategies for the management of heart failure patients at high risk for admissionA systematic review of

randomized trials. *Journal of the American College of Cardiology.* 2004;44(4):810-819.
66. Gilbody S, Whitty P, Grimshaw J, Thomas R. Educational and organizational interventions to improve the management of depression in primary care: A systematic review. *JAMA.* 2003;289(23):3145-3151.
67. Patwardhan MB, Kawamoto K, Lobach D, Patel UD, Matchar DB. Recommendations for a Clinical Decision Support for the Management of Individuals with Chronic Kidney Disease. *Clinical Journal of the American Society of Nephrology.* February 1, 2009 2009;4(2):273-283.
68. http://www.niddk.nih.gov/news/events-calendar/Pages/ckd-populations-2015.aspx. Accessed 12/1/2015.
69. Norman DA. *The psychology of everyday things.* New York: Basic Books; 1988.
70. Norman DA, Draper SW. *User centered system design : new perspectives on human-computer interaction.*
Hillsdale, N.J.: L. Erlbaum Associates; 1986.
71. Zhang J, Patel VL, Johnson TR, Shortliffe EH. A cognitive taxonomy of medical errors. *J Biomed Inform.* Jun 2004;37(3):193-204.
72. Stead WW, Lin H, National Research Council (U.S.). Committee on Engaging the Computer Science Research Community in Health Care Informatics., National Research Council (U.S.). Computer Science and Telecommunications Board., National Research Council (U.S.). Division on Engineering and Physical Sciences., ebrary Inc. *Computational technology for effective health care immediate steps and strategic directions.* Washington, D.C.: National Academies Press; 2009.

73. Medicine Io. *Crossing the Quality Chasm: A New Health System for the 21st Century* National Academy Press 2001.

74. Kidney Disease: Improving Global Outcomes (KDIGO) CKD Work Group. KDIGO clinical practice guideline for the evaluation and management of chronic kidney disease. *Kidney Int Suppl.* 2013;3:1-150.

75. O'Hare AM, Hotchkiss JR, Kurella Tamura M, et al. Interpreting treatment effects from clinical trials in the context of real-world risk information: end-stage renal disease prevention in older adults. *JAMA internal medicine.* Mar 1 2014;174(3):391-397.

76. Chapter 5: Referral to specialists and models of care. *Kidney International Supplements.* 1// 2013;3(1):112-119.

77. Lee B, Turley M, Meng D, et al. Effects of proactive population-based nephrologist oversight on progression of chronic kidney disease: a retrospective control analysis. *BMC Health Serv Res.* 2012;12(1):252.

78. Salgado TM, Moles R, Benrimoj SI, Fernandez-Llimos F. Pharmacists' interventions in the management of patients with chronic kidney disease: a systematic review. *Nephrology Dialysis Transplantation.* January 1, 2012 2012;27(1):276-292.

79. Stemer G, Lemmens-Gruber R. Clinical pharmacy activities in chronic kidney disease and end-stage renal disease patients: a systematic literature review. *BMC Nephrology.* 2011;12(1):35.

80. Gheewala P, Peterson G, Curtain C, Nishtala P, Hannan P, Castelino R. Impact of the Pharmacist Medication Review Services on Drug-Related Problems and Potentially Inappropriate Prescribing of Renally Cleared Medications in Residents of Aged Care Facilities. *Drugs & aging.* 2014/11/01 2014;31(11):825-835.

81. Nunes JW, Greene J, Wallston K, et al. Pilot Study of a Physician-Delivered Education Tool to Increase Patient Knowledge About CKD. *American journal of kidney diseases : the official journal of the National Kidney Foundation.* 03/27 2013;62(1):23-32.

82. Mason J, Khunti K, Stone M, Farooqi A, Carr S. Educational Interventions in Kidney Disease Care: A Systematic Review of Randomized Trials. *American Journal of Kidney Diseases.* 6// 2008;51(6):933-951.

83. Tangri N, Grams ME, Levey AS, et al. Multinational assessment of accuracy of equations for predicting risk of kidney failure: A meta-analysis. *JAMA.* 2016;315(2):164-174.

84. Waitman LR, Phillips IE, McCoy AB, et al. Adopting real-time surveillance dashboards as a component of an enterprisewide medication safety strategy. *Joint Commission journal on quality and patient safety / Joint Commission Resources.* Jul 2011;37(7):326-332.

85. Kovesdy CP, Coresh J, Ballew SH, et al. Past Decline Versus Current eGFR and Subsequent ESRD Risk. *J Am Soc Nephrol.* Dec 11 2015.

86. Fox CH, Vest BM, Kahn LS, et al. Improving evidence-based primary care for chronic kidney disease: study protocol for a cluster randomized control trial for translating evidence into practice (TRANSLATE CKD). *Implementation science : IS.* 2013;8:88.

87. Diamantidis CJ, Powe NR, Jaar BG, Greer RC, Troll MU, Boulware LE. Primary care-specialist collaboration in the care of patients with chronic kidney disease. *Clin J Am Soc Nephrol.* Feb 2011;6(2):334-343.

88. http://dashboard.healthit.gov/quickstats/pages/FIG-Vendors-of-EHRs-to-Participating-Professionals.php. Accessed November 5, 2016.

89. Epic. http://www.epic.com/. Accessed November 21, 2016.

90. http://dashboard.healthit.gov/quickstats/quickstats.php Accessed November 6, 2016.

91. Kosmisky DE, Alimi OR, Fitzgerald CR, et al. Student-pharmacist based medication

reconciliation to identify drug record discrepancies and medication-related problems in an outpatient hemodialysis unit. *Pharmacotherapy.* 2012;32(10):E295-E296.

92. Levey AS, Inker LA, Matsushita K, et al. GFR decline as an end point for clinical trials in CKD: a scientific workshop sponsored by the National Kidney Foundation and the US Food and Drug Administration. *Am J Kidney Dis.* Dec 2014;64(6):821-835.

93. Gibson OJ, Balami JS, Pope GA, Tarassenko L, Reckless IP. "Stroke Nav": A wireless data collection and review system to support stroke care delivery. *Comput Methods Programs Biomed.* Mar 6 2012.

94. Marshall S, Harrison J, Flanagan B. The teaching of a structured tool improves the clarity and content of interprofessional clinical communication. *Quality and Safety in Health Care.* April 1, 2009 2009;18(2):137-140.

95. Randmaa M, Mårtensson G, Leo Swenne C, Engström M. SBAR improves communication and safety climate and decreases incident reports due to communication errors in an anaesthetic clinic: a prospective intervention study. *BMJ open.* January 1, 2014 2014;4(1).

96. Panesar RS, Albert B, Messina C, Parker M. The Effect of an Electronic SBAR Communication Tool on Documentation of Acute Events in the Pediatric Intensive Care Unit. *American Journal of Medical Quality.* January 1, 2016 2016;31(1):64-68.

97. Pope BB, Rodzen L, Spross G. Raising the SBAR: how better communication improves patient outcomes. *Nursing.* Mar 2008;38(3):41-43.

98. Samal L, Linder JA, Bates DW, Wright A. Electronic problem list documentation of chronic kidney disease and quality of care. *BMC Nephrol.* 2014;15:70.

99. Levey AS, Stevens LA, Schmid CH, et al. A New Equation to Estimate Glomerular Filtration Rate. *Annals of internal medicine.* 2009;150(9):604-612.

100. Hemmelgarn BR, Manns BJ, Lloyd A, et al. Relation between kidney function, proteinuria, and adverse outcomes. *JAMA.* Feb 3 2010;303(5):423-429.

101. Hug BL, Witkowski DJ, Sox CM, et al. Occurrence of adverse, often preventable, events in community hospitals involving nephrotoxic drugs or those excreted by the kidney. *Kidney International.* 12/1/ 2009;76(11):1192- 1198.

102. Collaborators GBDRF, Forouzanfar MH, Alexander L, et al. Global, regional, and national comparative risk assessment of 79 behavioural, environmental and occupational, and metabolic risks or clusters of risks in 188 countries, 1990-2013: a systematic analysis for the Global Burden of Disease Study 2013. *Lancet.* Sep 10 2015.

103. Murray DM. *Design and analysis of group-randomized trials.* New York: Oxford University Press; 1998.

104. Ward BW, Schiller JS, Goodman RA. Multiple Chronic Conditions Among US Adults: A 2012 Update. *Preventing Chronic Disease.* 2014;11:E62.

105. Hootman JM, Helmick CG, Brady TJ. A public health approach to addressing arthritis in older adults: the most common cause of disability. *American journal of public health.* Mar 2012;102(3):426-433.

106. Yoon PW, Bastian B, Anderson RN, et al. Potentially preventable deaths from the five leading causes of death-- United States, 2008-2010. *MMWR. Morbidity and mortality weekly report.* May 2 2014;63(17):369-374.

107. Wagner EH, Austin BT, Davis C, Hindmarsh M, Schaefer J, Bonomi A. Improving Chronic Illness Care: Translating Evidence Into Action. *Health Affairs.* November 1, 2001 2001;20(6):64-78.

108. Wagner EH, Bennett SM, Austin BT, Greene SM, Schaefer JK, Vonkorff M. Finding common ground: patient- centeredness and evidence-based chronic illness care. *Journal of alternative and complementary medicine.* 2005;11 Suppl 1:S7-15.
