# Supplementary material for: Electronic Health Record Population Health Management for Chronic Kidney Disease Care: A Cluster Randomized Clinical Trial
Source: JAMA Intern Med. 2024 Apr 15;184(7):737–47. doi: 10.1001/jamainternmed.2024.0708 (PMC11019443; doi:10.1001/jamainternmed.2024.0708)
Supplement: Supplement 2. — Statistical analysis plan [file jamainternmed-e240708-s002.pdf]

Statistical Analysis Plan

Original Statistical Analysis Plan (from grant application)

Clinical outcome (primary study outcome): A ≥40% decline in eGFR or ESRD.(1) eGFR decline will be adjudicated based on the baseline creatinine and eGFR determined from the CKD-EPI equation and measured routinely in clinical practice.(2) To limit ascertainment bias, we will also use a once yearly decision support alert to remind study PCPs in both arms to order a BMP on study patients, if results are not available in the last 6 months. ESRD will be defined as an eGFR < 10ml/min to account for patients with markedly reduced baseline eGFR values (i.e., 16-20ml/min). To limit surveillance bias, in addition to the above alert, we will use 6-month ascertainment windows and average all values within each window. Additional analyses will compare changes in eGFR slope over time (using splines to account for non-linearity). Researchers involved in outcome assessment will be strictly blinded.

Process of care outcomes (secondary outcomes): 1) HTN control. Outpatient, sitting BP values measured during each outpatient encounter and recorded in the EHR. BP will be treated as a continuous variable. To minimize ascertainment bias, we will use 6-month ascertainment windows to determine an average BP for each patient for each 6-mo period. Patients lacking an outpatient value will have their last value carried forward. 2) Use of RAASi. Will be determined by active use of an ACEi or ARB based on the EHR medication list at each outpatient encounter. Analyses will compare cumulative person-time exposure during the study. 3) Medication safety. We will examine the rates of use of several high-risk medications that can be associated with adverse outcomes in progressive CKD. Medication exposure will be determined by presence of the specified medication on the patient's EHR medication list at each outpatient encounter. Analyses will compare cumulative person-time exposure during the study. a) Use of NSAIDs: use examined for all study patients, b) Use of glyburide: use examined for all diabetic study patients, c) Use of metformin: use examined for diabetic study patients with eGFR<30, d) Use of gemfibrozil: use examined for all study patients with eGFR<30.

Sample Size: We base our sample size justifications on computational techniques that match our study design and proposed analytical approach within the constraints of published methodologies (PASS 13 Power Analysis and Sample Size Software (2014). NCSS, LLC. Kaysville, Utah, USA). We selected our sample size to attain adequate power to assess differences in the primary outcome using the design effect method for time-to-event approach.(3) We assumed two-sided tests at $\alpha = 0.05$, an Intra-Class Correlation (ICC) of 0.01 (as recommended for health services research when no preliminary data on ICC are available)(4), a cluster size of 19 patients per practice, and an 18-month enrollment period with an additional follow-up of 24 months after the accrual period. For an individually randomized trial, a total sample size of 1,102 provides at least 80% power to detect a hazard ratio of 0.64 (or a 0.05 difference in event proportions) assuming the control event proportion at 24 months is 0.15, based on our preliminary data. Accounting for the clustered design and 20% attrition (e.g., patients leaving the health system), the required sample size is 1,653.
For the secondary outcome (BP in patients with HTN and total person-time of medication exposures), this sample size achieves at least 80% power to detect a small effect size, standardized mean difference of at least 0.2. In the 96% of the cohort estimated to have a diagnosis of HTN, this is equivalent to detecting a mean SBP difference of 3mmHg (based on preliminary data with SBP standard deviation [SD] = 15mmHg), a mean RAASi use difference of ~78 person-years (modeled SD = 390 person-years, based on simulation), a mean medication

use exposure difference of 72 person-years (modeled SD= 360 person-years based on simulation).

Preliminary Analyses: Preliminary analyses will focus on data checks for completeness and accuracy and address any issues with data quality. Descriptive summaries will be examined overall and by intervention group and time point. We will compare distributions of baseline characteristics for practices and patients between randomized groups to assess the effectiveness of randomization. All primary analyses for intervention group comparisons will use an intention-to-treat approach and results will be reported using the CONSORT extension to cluster RCTs.(5) We will adjust for statistical or clinical differences in secondary analyses.

General Approach: We will use linear mixed models (LMM) or generalized LMM (GLMM) to account for clustering. These models will include random practice intercepts to account for correlation of observations from patients within the same practice. For analysis involving repeated measurements, random patient intercepts nested within practice effects will also be included. Unadjusted models will test a fixed intervention effect; adjusted models may include stratification variables used in randomization, patient and practice characteristics exhibiting imbalance between intervention groups, and variables associated with missingness.

Primary clinical outcome (Aim 2: eGFR decline ≥40% or ESRD): Our primary analysis will use discrete-time survival methods to examine the occurrence of the composite endpoint at 6 month intervals from baseline. At each of these discrete time points, the average of all eGFR measurements within a +/- 3 month window will be used to determine event occurrence. This accounts for random eGFR fluctuations and will minimize the impact of potential ascertainment bias related to more frequent eGFR measurements in the intervention group. We will use a GLMM for binary outcomes with complementary log-log link and piecewise-constant hazards. This model will include random practice intercepts to account for practice-level clustering. This is analogous to a Cox model with frailty for continuous-time survival data (i.e., a random effects survival analysis model). As a secondary analysis, we will treat eGFR as a continuous variable and compare the rate of decline over time between the intervention and control group using GLMM with an identity link under the normal family. This will utilize all repeated outpatient eGFRs from each patient. Random patient intercepts nested within random practice intercepts will be included to account for within patient and within practice correlations. The unadjusted model will include fixed effects for intervention, time, and treatment by time interaction. We will test for significance of the treatment by time interaction to test intervention effects. As sensitivity analyses, we will a) fit smoothing-spline mixed-effects models since eGFR trajectories may be nonlinear, and b) require 2 consecutive eGFR values below the 40% decline/ESRD threshold.

Secondary process of care outcomes (Aim 1): To assess the intervention effect on HTN control, we will compare mean SBP between intervention and control at each 6-month time point using LMM with random practice intercepts. At each of these time points, the average of all BP measurements within a +/- 3 month window will be used to account for random fluctuations. As an alternative approach, we will analyze BP as a binary outcome defined by achieving BP goal of <140/90 via GLMM with logit link and binomial family. In examining medications (e.g., RAASi), we will calculate the total number of medication days for each patient. The average medication duration will be compared between intervention and control using LMM with random practice effect.

Exploratory analyses: Although this study is not powered to conduct subgroup analyses, we will perform analyses of the secondary outcomes stratified by DM status, HTN with baseline BP (>140/90), and RAASi use to explore whether heterogeneous intervention effects exist among these subgroups.

Missing Data: The extent of missing data will be described. We will investigate the randomness of missing data using available information on patient and provider characteristics to identify possible covert missing data mechanisms. The analytical models used can handle data that are missing at random, but other strategies to handle missing data (multiple imputation, selection models, pattern-mixture models) will also be implemented. In addition, adjusted LMM or GLMM will be used to account for variables associated with missingness.

Final Statistical Analysis Plan

Clinical outcome (primary study outcome): A ≥40% decline in eGFR or ESRD.(1) eGFR decline will be adjudicated based on the baseline creatinine and eGFR determined from the CKD-EPI creatinine (2021) equation.(6). To limit ascertainment bias, we will also use a once yearly decision support alert to remind study PCPs in both arms to order a BMP on study patients, if results are not available in the last 6 months. ESRD will be defined as an eGFR < 10ml/min to account for patients with markedly reduced baseline eGFR values (i.e., 16-20ml/min). To limit surveillance bias, in addition to the above alert, we will use 6-month ascertainment windows and average all values within each window. (Table 1). Researchers involved in outcome assessment will be strictly blinded.

Process of care outcomes (secondary outcomes): 1) HTN control. Outpatient, sitting BP values measured during each outpatient encounter and recorded in the EHR. BP will be treated as a continuous variable. To minimize ascertainment bias, we will use 6-month ascertainment windows to determine an average BP for each patient for each 6-mo period. 2) Use of RAASi. Will be determined by active use of an ACEi or ARB based on the EHR medication list at each outpatient encounter. Analyses will compare cumulative person-time exposure during the study. 3) Medication safety. We will examine the rates of use of several high-risk medications that can be associated with adverse outcomes in progressive CKD. Medication exposure will be determined by presence of the specified medication on the patient's EHR medication list at each outpatient encounter. Analyses will compare cumulative person-time exposure during the study. a) Use of NSAIDs: use examined for all study patients, b) Use of glyburide: use examined for all diabetic study patients, c) Use of metformin: use examined for diabetic study patients with eGFR<30, d) Use of gemfibrozil: use examined for all study patients with eGFR<30.

Sample Size: We base our sample size justifications on computational techniques that match our study design and proposed analytical approach within the constraints of published methodologies (PASS 13 Power Analysis and Sample Size Software (2014). NCSS, LLC. Kaysville, Utah, USA). We selected our sample size to attain adequate power to assess differences in the primary outcome using the design effect method for time-to-event approach.(3) We assumed two-sided tests at $\alpha = 0.05$, an Intra-Class Correlation (ICC) of 0.01 (as recommended for health services research when no preliminary data on ICC are available)(4), a cluster size of 19 patients per practice, and an 18-month enrollment period with an additional follow-up of 24 months after the accrual period. For an individually randomized trial, a total sample size of 1,102 provides at least 80% power to detect a hazard ratio of 0.64 (or a 0.05 difference in event proportions) assuming the control event proportion at 24 months is 0.15, based on our preliminary data. Accounting for the clustered design and 20% attrition (e.g., patients leaving the health system), the required sample size is 1,653.
For the secondary outcome (BP in patients with HTN and total person-time of medication exposures), this sample size achieves at least 80% power to detect a small effect size, standardized mean difference of at least 0.2. In the 96% of the cohort estimated to have a diagnosis of HTN, this is equivalent to detecting a mean SBP difference of 3mmHg (based on preliminary data with SBP standard deviation [SD] = 15mmHg), a mean RAASi use difference of ~78 person-years (modeled SD = 390 person-years, based on simulation), a mean medication

use exposure difference of 72 person-years (modeled SD= 360 person-years based on simulation).

Preliminary Analyses: Preliminary analyses will focus on data checks for completeness and accuracy and address any issues with data quality. Descriptive summaries will be examined overall and by intervention group and time point. We will compare distributions of baseline characteristics for practices and patients between randomized groups to assess the effectiveness of randomization. All primary analyses for intervention group comparisons will use an intention-to-treat approach and results will be reported using the CONSORT extension to cluster RCTs.(5) We will adjust for statistical or clinical differences in secondary analyses.

General Approach: We will use linear mixed models (LMM) or generalized LMM (GLMM) to account for clustering. These models will include random practice intercepts to account for correlation of observations from patients within the same practice. For analysis involving repeated measurements, random patient intercepts nested within practice effects will also be included. Unadjusted models will test a fixed intervention effect; adjusted models will include stratification variable used in randomization (practice size) and patient characteristics (age, sex, race, baseline levels). All statistical analyses will be performed in R using glmmTMB and lme4 packages to fit discrete-time survival models, GLMM, and LMM and use the marginaleffects package to estimate adjusted average responses and contrasts.

Primary clinical outcome (Aim 2: eGFR decline ≥40% or ESRD): Our primary analysis will use discrete-time survival methods to examine the occurrence of the composite endpoint at 6-month intervals from baseline. To determine progression at each of these discrete time points, the average of all eGFR measurements within a +/- 3-month window will be used in order to account for random eGFR fluctuations and potential ascertainment bias. We will employ generalized GLMM with complementary log-log link with random practice intercepts to account for practice-level clustering. This is analogous to a Cox model with frailty for continuous-time survival data (i.e., a random effects survival analysis model). The unadjusted model will include fixed effects for intervention, and time. The functional form of time (categorical or continuous with restricted cubic splines) will be selected based on the Akaike Information Criteria (AIC). Adjusted models will include pre-specified patient (age, sex, race, baseline eGFR) and practice variables (practice size). MMWD, being moved to hospice care, or mortality will be treated as competing events. Those that did not reach any endpoint will be censored at the end of study (July 31, 2022). In secondary analysis, we will use eGFR as a continuous variable and include random patient intercepts nested within random practice intercepts. The unadjusted model included fixed effects for intervention, time, and treatment by time interaction. We will compare the rate of eGFR decline over time between groups. As sensitivity analyses, we evaluated a) smoothing-spline mixed-effects models since eGFR trajectories may be nonlinear, and b) requiring 2 consecutive eGFR values below the 40% decline/ESKD threshold.

Secondary process of care outcomes (Aim 1): To assess the intervention effect on hypertension control, we will compare the mean outpatient systolic blood pressure (SBP) between intervention and control at each 6-month time point using LMM with fixed effects for treatment, time, and treatment by time interaction. At each time point, the average of all BP measurements within a +/- 3-month window will be used to account for random fluctuations. As an alternative approach, we will analyze BP as a binary outcome defined by achieving BP goal of <140/90 mm Hg or <130/80 mm Hg via GLMM with logit link and binomial family. If linearity of time is reasonable, we will compare the slopes between intervention and control. These models will include random patient intercepts nested within random practice intercepts and will be adjusted for pre-specified covariates (age, sex, race, and practice size). In examining medication use, we will compare the average medication exposure days between the arms using LMM with random practice effect. For each medication class, the number of exposure days will be determined by counting the number of days from medication order start date to

either the discontinuation date, survival endpoint date (progression, ESKD, death/hospice, MMWD) or study end date, whichever comes first. (refer to Table 1 for details). To account for potential overdispersion, we will also fit count models including Poisson, generalized Poisson, and negative binomial mixed models and select the final model based on minimum Akaike Information Criteria (AIC) or Bayesian Information Criteria (BIC). If count models provide better fit, we will report the rate of exposure days per year for each medication. We will adjust for baseline medication exposure days in addition to pre-specified covariates with the log of the number follow-up days as offset. In post-hoc analyses, we will evaluate the effect of intervention on albuminuria minimization among patients with at least one urine-albumin-to-creatinine ratio (UACR) measure during the study period. Similar to the approach we used for examining changes in eGFR, we will fit a mixed effects model utilizing all available UACR for each patient adjusting for age, sex, race, baseline eGFR and practice size. We will also adjust for diabetes since we found that it is associated with having at least one UACR measure during the study period. Changes in UACR from baseline to 18 months (approximately the median follow-up time) were calculated by group, and between group differences were compared using contrasts.

Exploratory analyses: Although this study is not powered to conduct subgroup analyses, we will perform analyses stratified by age, sex, CKD stage, DM status, HTN with baseline BP (>140/90 mmHg or >130/80 mmHg), and ACEi/ARB in albuminuric patients, or SGLT-2i use to explore whether heterogeneous intervention effects exist among these subgroups. Subgroup by race will also be examined.

Missing Data: The extent of missing data will be described. We will investigate the randomness of missing data using available information on patient and provider characteristics to identify possible covert missing data mechanisms. The analytical models used can handle data that are missing at random, but other strategies to handle missing data (multiple imputation, selection models, pattern-mixture models) will also be implemented. In addition, adjusted LMM or GLMM will be used to account for variables associated with missingness.

Table 1. Determination of outcomes and covariates based from electronic health record (EHR)

	**Determination from EHR**
**General principles**	
Baseline time point (T0)	• Office or telemedicine encounters with the PCP after the date the patient was determined to be eligible will be extracted. The first PCP encounter within 1 year of the date of eligibility determination will be used as the baseline time point (T0).
Outpatient records	• Outpatient data will be extracted from the EHR. Laboratory results or vital records obtained within a span of 2 consecutive days will be excluded, as these are more likely to stem from inpatient encounters.
**Primary outcome**	
Composite of ≥40% decline in eGFR from baseline or ESKD	• The CKD-EPI creatinine (2021) equation will be used to calculate eGFR. (6)<br>• The baseline eGFR will be calculated by averaging the two most recent serum creatinine values, which should be at least 90 days apart, within a 3-year look-

	back period from the first PCP visit (T0). Improbable creatinine values (>10) will be excluded. If a second creatinine measurement beyond 90 days is not available, we will progressively reduce the time requirement to at least 60 days and then at least 30 days. In cases where a second creatinine value cannot be found, the single closest creatinine value to T0 will be utilized.<br>• eGFR at follow-up will be determined by averaging all eGFR measurements within +/- 3 months from the landmark time points (6, 12, 18, and 24 months).<br>• Progression or ESKD flags will be generated at each follow-up time point. Progression will be identified if the averaged eGFR at the respective time point is ≥ 40% of the baseline eGFR. ESKD will be recognized if the averaged eGFR at the corresponding time point is <10 ml/min, or if ICD-10 or CPT codes indicating ESKD or kidney transplant are detected within the time window of that specific time point.<br>• At each index time point, endpoints for MMWD and hospice/death will be determined based on ICD-10 codes or the date of death within the specified time window.<br>• Individuals who do not reach any endpoint will be considered censored at the conclusion of the study (July 31, 2022). Patients with no EHR records at the follow-up point will be presumed not to have reached any endpoint and will also be censored.
Secondary outcomes	
eGFR (continuous)	• Each follow-up eGFR, calculated using the CKDI-EPI (2021), will be used without averaging. Elapsed time from T0 will be determined based on the date the creatinine was drawn.
Composite of confirmed progression or ESKD	• For the purpose of sensitivity analyses, we will consider progression confirmed if the subsequent eGFR measurement, taken at least a month after the initial progression, continues to show a decline of ≥40% from the baseline value. The composite outcome is determined using a similar approach to the primary outcome.
Blood pressure (continuous)	• Baseline BP will be determined by taking the most recent systolic (SBP) and diastolic BP (DBP) prior to T0.

	• BP at follow-up will be determined by averaging all BP measurements within +/- 3 months of the landmark time points (6, 12, 18, and 24 months).<br>• Implausible BP values (systolic BP<70 or >250; diastolic BP<10 or >180) will be excluded.
Hypertension control	• Hypertension control will be defined as SBP<140 and DBP<90. Alternative definition with SBP<130 and DBP<80 will also be used to reflect more recent guidelines.<br>• Continuous BP measurements described above will be used to determine hypertension control at baseline and at each of the landmark time points (6, 12, 18, and 24 months).
Medication use and exposure days	• Medication list will be extracted from EHR medication orders that were flagged as sent. Subcutaneous and oral medications were excluded.<br>• Medications will be classified into ACEi/ARB, NSAID, Glyburide, Metformin, and Gemfibrozil. Use of insulin, SGLT2, GLP1, and moderate to high intensity statin will also be determined.<br>• For each medication class, the count of exposure days will be determined by calculating the duration from the medication order start date up to the earlier of the discontinuation date, survival endpoint date (progression, ESKD, death/hospice, MMWD), or the study end date. In the case of chronic medications, if the discontinuation date is unknown, an expiration date of one year from the start date will be assumed. For NSAIDs, exposure days will be computed based on medication refills, as these are not consistently prescribed for extended periods. In situations involving multiple medication episodes, the cumulative exposure days across all episodes will be utilized.<br>• At baseline, a medication will be considered active if the start date was within 1 year prior to T0 and has not been discontinued by T0.
**Other covariates**	
Urine albuminuria	• The most recent urine albumin and urine creatinine within 2-years prior to T0 will be used to calculate urine albumin-to-creatinine ratio (UACR). If this is missing, it will be estimated from protein quantification using conversion formulas for urine

	protein-creatinine ratio or urine dipstick protein.(7)
Kidney failure risk equation (KFRE)	• At baseline, 5-year risk and 2-year risk of ESKD will be calculated from baseline eGFR and UACR using a validated 4-variable KFRE.(8)<br>• A 5 year KFRE ≥ 4% will be considered high-risk CKD.
Laboratory values	• K+, hemoglobin, albumin, hemoglobin A1c<br>• Baseline laboratory values will be determined by averaging the two most recent lab measurements within 1 year prior to T0.<br>• Implausible lab values were excluded (hemoglobin≥27; hemoglobin A1c>20) or censored (albumin<1 censored at 1).
Comorbid conditions	• Diabetes (type 1 and type 2), hypertension, hyperlipidemia, coronary artery disease, cerebrovascular disease, peripheral vascular disease, congestive heart failure, arrhythmia, gout, chronic lung disease, chronic liver disease, mood disorder, malignancy, Charlson comorbidity score.<br>• Comorbid conditions will be identified based on ICD-9 or ICD10 records, requiring at least 1 incidence in the problem list or 2 incidences from the diagnosis list.<br>• Baseline values will be defined on the date of first PCP visit (T0).
Sodiodemographics	• age, sex, race, ethnicity, marital status, median income at zip code of residence, area deprivation index, Rural-Urban category (RUCA score), BMI<br>• Baseline values will be defined on the date of first PCP visit (T0).<br>• For BMI, the most recent height and weight measurements prior to T0 will be used in the calculation.
Care utilization	• The number of visits to the PCP, cardiologist, ER, and hospitalizations at baseline will be determined using a 1 year look-back period from T0.

Summary of Major Revisions to the SAP

The major changes in the SAP and their underlying reasoning are itemized below. These determinations were made prior to the initial disclosure of the study outcomes to the research team in March 2023.

- Establishment of Baseline Date: The baseline date was set as the first primary care provider (PCP) visit within one year of the eligibility determination date. This choice was made to ensure a common starting point (time 0) for both study arms, which is crucial for conducting time-to-event analyses.
- eGFR Calculation: To align with current clinical practice, the CKD-EPI 2021 equation was adopted for eGFR calculations.
- Competing Events: Events such as Medication Management Without Dialysis (MMWD), transition to hospice care, or mortality were considered as competing events as these endpoints may informatively censor the primary outcome (progression or ESKD). An exploration of the intervention's impact on mortality and the composite of progression, ESKD, or mortality was planned.
- Adjusted Models: Efficiency enhancement was pursued through the inclusion of pre-specified covariates in adjusted models: age, sex, race, and baseline eGFR. These factors were anticipated to be linked with the primary outcome.
- Subgroup Analyses: Subgroups were formed based on age, sex, and CKD stage. The aim was to scrutinize whether treatment effects differed across diverse patient demographic and clinical characteristics.
- SAP Layout and Detail Enhancement: The SAP's layout was restructured, and more comprehensive details about variable extraction from EHR were added.

Further alterations were enacted following the unveiling of the initial study results to the research team in March 2023.

- Hypertension Control Definition: To remain consistent with contemporary guidelines, a blood pressure (BP) goal of <130/80 mm Hg (SBP/DBP) was integrated as an alternate hypertension control endpoint. This definition was also added to subgroup analyses related to the primary outcome.
- Medication Exposure Days Calculation: To limit missing data in medication exposure days calculation, chronic medications were assumed to be active for one year from the medication start date when discontinuation date is unknown. For NSAIDs, medication refills were used as these are not consistently prescribed for extended periods.
- Model Considerations for Count Data: Considering the skewed nature of the data, models for count data were explored for the analysis of medication exposure days. The final model was determined based on AIC and BIC criteria.
- Intervention effect on UACR: Following the suggestion of a reviewer, we examined the effect of the intervention on albuminuria minimization by analyzing the between group changes in UACR.
- Race subgroup: To address a reviewer’s concern about representativeness of the population with respect to race, we performed subgroup analysis by race.

References

1. Levey AS, Inker LA, Matsushita K, Greene T, Willis K, Lewis E, et al. GFR decline as an end point for clinical trials in CKD: a scientific workshop sponsored by the National Kidney Foundation and the US Food and Drug Administration. Am J Kidney Dis Off J Natl Kidney Found. 2014 Dec;64(6):821–35.
2. Levey AS, Stevens LA, Schmid CH, Zhang YL, Castro AF, Feldman HI, et al. A new equation to estimate glomerular filtration rate. Ann Intern Med. 2009 May 5;150(9):604–12.
3. Rutterford C, Copas A, Eldridge S. Methods for sample size determination in cluster randomized trials. Int J Epidemiol. 2015 Jun;44(3):1051–67.
4. Thompson DM, Fernald DH, Mold JW. Intraclass Correlation Coefficients Typical of Cluster-Randomized Studies: Estimates From the Robert Wood Johnson Prescription for Health Projects. Ann Fam Med. 2012 May;10(3):235–40.
5. Schulz KF, Altman DG, Moher D, CONSORT Group. CONSORT 2010 statement: updated guidelines for reporting parallel group randomised trials. BMJ. 2010 Mar 23;340:c332.
6. Inker LA, Eneanya ND, Coresh J, Tighiouart H, Wang D, Sang Y, et al. New Creatinine- and Cystatin C-Based Equations to Estimate GFR without Race. N Engl J Med. 2021 Nov 4;385(19):1737–49.
7. Sumida K, Nadkarni GN, Grams ME, Sang Y, Ballew SH, Coresh J, et al. Conversion of urine protein-creatinine ratio or urine dipstick to urine albumin-creatinine ratio for use in CKD screening and prognosis: An individual participant-based meta-analysis. Ann Intern Med. 2020 Sep 15;173(6):426–35.
8. Tangri N, Stevens LA, Griffith J, Tighiouart H, Djurdjev O, Naimark D, et al. A Predictive Model for Progression of Chronic Kidney Disease to Kidney Failure. JAMA. 2011 Apr 20;305(15):1553–9.
