# Supplementary material for: Electronic Health Record Population Health Management for Chronic Kidney Disease Care: A Cluster Randomized Clinical Trial
Source: JAMA Intern Med. 2024 Apr 15;184(7):737–47. doi: 10.1001/jamainternmed.2024.0708 (PMC11019443; doi:10.1001/jamainternmed.2024.0708)
Supplement: Supplement 3. — Grant proposal plan [file jamainternmed-e240708-s003.pdf]

Socio-demographics	• Age, gender, race, ethnicity, marital status, insurance, and zip code for linkage with neighborhood median household income.[39]<br>• Baseline values defined on the date of baseline visit	EHR
Comorbid conditions	• DM, HTN, hyperlipidemia, CAD, cerebrovascular disease, peripheral vascular disease, CHF, arrhythmia, gout, chronic lung disease, chronic liver disease, mood disorder, and malignancy.<br>• Baseline values defined on the date of baseline visit and using a 24 month "look back" period.	Phenotypes using administrative & clinical codes, meds, & lab values validated in local EHR.[12,38,39]
Blood Pressure	• Baseline BP - mean outpatient BP from the date of the baseline study visit with the PCP until 180 days prior to the baseline visit.<br>• Follow-up BP – all outpatient BPs after patient enrollment	Office visit vital signs recorded in the EHR
Medication use	• RAASi, NSAID and other medications deemed a potential safety concern (e.g., allopurinol, gemfibrozil, glyburide, metformin, etc.)<br>• Medication related problems and drug record discrepancies	EHR medication list; medication review (intervention patients)
Laboratory values	• Common laboratory tests (e.g., *K+*, cholesterol, etc.)<br>• Baseline values - determined using the most recent value from baseline visit up to 365 days prior to the visit.	EHR (restricted to outpatient labs)
Urine albuminuria	• Quantitative urine albuminuria - most recent ACR from the baseline visit up to 365 days prior to the visit.<br>• Urine dipstick albuminuria - median of outpatient values available from the date of baseline visit up to 365 days prior to the visit.[100]	Outpatient lab values from EHR

C. 2.2.10.a. Aim 1: Process of care outcomes (secondary outcomes).
1) HTN control. Outpatient, sitting BP values measured during each outpatient encounter and recorded in the EHR. BP will be treated as a continuous variable. To minimize ascertainment bias, we will use 6-month ascertainment windows to determine an average BP for each patient for each 6-mo period. Patients lacking an outpatient value will have their last value carried forward (see C.2.2.12). Preliminary data indicate >93% of high-risk patients had a new outpatient BP value every 6-months over a 36-month interval.

2) Use of RAASi. Will be determined by active use of an ACEi or ARB based on the EHR medication list at each outpatient encounter. Analyses will compare cumulative person-time exposure during the study.

3) Medication safety. We will examine the rates of use of several high-risk medications[21,43,54,61,80,101] that can be associated with adverse outcomes in progressive CKD. Medication exposure will be determined by presence of the specified medication on the patient's EHR medication list at each outpatient encounter. Analyses will compare cumulative person-time exposure during the study.
a) Use of NSAIDs: use examined for all study patients, b) Use of glyburide: use examined for all diabetic study patients, c) Use of metformin: use examined for diabetic study patients with eGFR<30, d) Use of gemfibrozil: use examined for all study patients with eGFR<30.

C. 2.2.10.b. Aim 2: Clinical outcome (primary study outcome). A $\geq$40% decline in eGFR or ESRD.[92] eGFR decline will be adjudicated based on the baseline creatinine and eGFR determined from the CKD-EPI equation and measured routinely in clinical practice.[99] *To limit ascertainment bias, we will also use a once yearly decision support alert to remind study PCPs in both arms to order a BMP on study patients, if results are not available in the last 6 months.* ESRD will be defined as an eGFR $\leq$ 10ml/min to account for patients with markedly reduced baseline eGFR values (i.e., 16-20ml/min).

The 40% decline surrogate outcome may increase power and precision by capturing additional events while maintaining a similar risk of type I error[92] compared to the standard doubling of serum creatinine outcome. To limit surveillance bias, in addition to the above alert, we will use 6-month ascertainment windows and average all values within each window. Our data indicate 75% of high-risk patients have an outpatient eGFR value every 6-months. Additional analyses will compare changes in eGFR slope over time (using splines to account for non-linearity, see C.2.2.12). Researchers involved in outcome assessment will be strictly blinded.

C.2.2.10.c. Exploratory outcomes. Mortality, *hyperkalemia*, and health utilization *(i.e., costs)* including hospitalizations, emergency department visits, and outpatient encounters will be ascertained by a combination of EHR and administrative data from the health plan (see LOS). The accessibility of administrative data that captures events outside the health system and supplements the EHR is a unique and complementary resource that will be leveraged in future study analyses. *Annual safety reviews will also examine these key outcomes*.

C.2.2.11. **Analytic Approach**. Preliminary analyses will focus on data checks for completeness and accuracy

and address any issues with data quality. Descriptive summaries will be examined overall and by intervention group and time point. We will compare distributions of baseline characteristics for practices and patients between randomized groups to assess the effectiveness of randomization. All primary analyses for intervention group comparisons will use an *intention-to-treat* approach and results will be reported using the CONSORT extension to cluster RCTs.[102] We will adjust for statistical or clinical differences in secondary analyses.

General approach: We will use linear mixed models (LMM) or generalized LMM (GLMM) to account for clustering. These models will include random practice intercepts to account for correlation of observations from patients within the same practice. For analysis involving repeated measurements, random patient intercepts nested within practice effects will also be included. Unadjusted models will test a fixed intervention effect; adjusted models may include stratification variables used in randomization, patient and practice characteristics exhibiting imbalance between intervention groups, and variables associated with missingness.

Primary clinical outcome (Aim 2: eGFR decline >40% or ESRD). Our primary analysis will use discrete-time survival methods to examine the occurrence of the composite endpoint at 6 month intervals from baseline. At each of these discrete time points, the average of all eGFR measurements within a +/- 3 month window will be used to determine event occurrence. This accounts for random eGFR fluctuations and will minimize the impact of potential ascertainment bias related to more frequent eGFR measurements in the intervention group. We will use a GLMM for binary outcomes with complementary log-log link and piecewise-constant hazards. This model will include random practice intercepts to account for practice-level clustering. This is analogous to a Cox model with frailty for continuous-time survival data (i.e., a random effects survival analysis model). As a secondary analysis, we will treat eGFR as a continuous variable and compare the rate of decline over time between the intervention and control group using GLMM with an identity link under the normal family. This will utilize all repeated outpatient eGFRs from each patient. Random patient intercepts nested within random practice intercepts will be included to account for within patient and within practice correlations. The unadjusted model will include fixed effects for intervention, time, and treatment by time interaction. We will test for significance of the treatment by time interaction to test intervention effects. As sensitivity analyses, we will a) fit smoothing-spline mixed-effects models since eGFR trajectories may be nonlinear,[102] and b) require 2 consecutive eGFR values below the 40% decline/ESRD threshold.

Secondary process of care outcomes (Aim 1): To assess the intervention effect on HTN control, we will compare mean SBP between intervention and control at each 6-month time point using LMM with random practice intercepts. At each of these time points, the average of all BP measurements within a +/- 3 month window will be used to account for random fluctuations. As an alternative approach, we will analyze BP as a binary outcome defined by achieving BP goal of <140/90 via GLMM with logit link and binomial family. In examining medications (e.g., RAASi), we will calculate the total number of medication days for each patient. The average medication duration will be compared between intervention and control using LMM with random practice effect. *Exploratory analysis:* Although this study is not powered to conduct subgroup analyses, we will perform analyses of the secondary outcomes stratified by DM status, HTN with baseline BP (>140/90), and RAASi use to explore whether heterogeneous intervention effects exist among these subgroups.

Missing Data: The extent of missing data will be described. We will investigate the randomness of missing data using available information on patient and provider characteristics to identify possible covert missing data mechanisms. The analytical models used can handle data that are missing at random, but other strategies to handle missing data (multiple imputation, selection models, pattern-mixture models) will also be implemented. In addition, adjusted LMM or GLMM will be used to account for variables associated with missingness.

Sample Size: We base our sample size justifications on computational techniques that match our study design and proposed analytical approach within the constraints of published methodologies (PASS 13 Power Analysis and Sample Size Software (2014). NCSS, LLC. Kaysville, Utah, USA). We selected our sample size to attain adequate power to assess differences in the primary outcome using a time-to-event approach.[102] We assumed two-sided tests at $\alpha = 0.05$, an Intra-Class Correlation (ICC) of 0.01 (as recommended for health services research when no preliminary data on ICC are available),[103] a cluster size of 19 patients per practice, and an 18-month enrollment period with an additional follow-up of 24 months after the accrual period. For an individually randomized trial, a total sample size of 1,102 provides at least 80% power to detect a hazard ratio of 0.64 (or a 0.05 difference in event proportions) assuming the control event proportion at 24 months is 0.15, based on our preliminary data. Accounting for the clustered design and 20% attrition (e.g., patients leaving the health system), the required sample size is 1,653. We propose to enroll 1,700 patients to be conservative.

For the secondary outcome (BP in patients with HTN and total person-time of medication exposures), this sample size achieves at least 80% power to detect a small effect size, standardized mean difference of at least 0.2. In the 96% of the cohort estimated to have a diagnosis of HTN, this is equivalent to detecting a mean SBP difference of 3mmHg (based on preliminary data with SBP standard deviation [SD] = 15mmHg), a mean RAASi

use difference of ~78 person-years (modeled SD = 390 person-years, based on simulation), a mean medication use exposure difference of 72 person-years (modeled SD= 360 person-years based on simulation).

C. 2.2.12. Anticipated results and interpretation. We anticipate the intervention will decrease rates of the primary clinical outcome (i.e., CKD progression) and will also improve HTN control, RAASi use, and medication safety (i.e., secondary outcomes). Slowing CKD progression through PHM while transforming access to subspecialist guidance would indicate that pragmatic, EHR-based interventions targeting high-risk patients can improve clinically salient outcomes. Future work would examine the cost-effectiveness of the intervention, the minimal elements required to meaningfully improve CKD patient care, safety, and outcomes; the physician and practice level predictors that underlie successful implementation, transportability to lower resourced settings, and refinements in patient markers of high-risk status. Our intervention would also serve as a model for other chronic diseases, which affect over ½ the population[104] and are leading causes of death and disability.[105,106]

If the study demonstrates improvements in clinical outcomes but no change in processes of care, then we will use the administrative and EHR data to examine novel differences in care delivery between arms that may have led to improvements. Alternatively, if the study demonstrates improvements in processes of care in the intervention arm but not in the composite clinical outcome, then we will explore whether extended follow-up may demonstrate improvements in clinical outcomes. Further, we will leverage the EHR and administrative data to explore whether additional, poorly characterized, unaddressed gaps in care could substantially contribute to CKD progression and mask the anticipated clinical improvements.

If there is no difference between the intervention and control groups, we will explore heterogeneity in outcomes related to practice and provider characteristics including patient volume, length of visits, etc. and conduct qualitative assessments to examine barriers/facilitators to implementation. The study would continue to provide critical information on heterogeneity of effects, CKD phenotypes, and refinements to CKD predictive models. These deliverables would strengthen future trials.

C.2.2.13. Potential Pitfalls and Alternative Approaches.
a) *Busy PCPs may not respond to EHR messages and embedded decision support*: We anticipate buy-in will be high given our provider centric approach to hone the intervention and maximize acceptability. We have also aligned with UPMC quality metrics to provide financial incentives. We will assess non-response and declination rates for the intervention by practice. We will reach out to groups with low participation rates to obtain feedback in a timely fashion to determine whether novel workflow challenges are present (e.g., including an additional EHR alert to target providers during instead of before their clinic visits). Further tailoring of the workflow will be undertaken to ensure efficiency for each practice when necessary. In addition, our high-level and lead provider support can be leveraged to ensure that study participation is highlighted and low users are re-engaged.

b) *PCPs may choose to primarily refer patients for traditional nephrology office consultations*: This would still result in more timely identification and management of high-risk CKD patients (vs. controls). However, PCPs have said that many high-risk patients are referred late due to the inconvenience of additional provider visits.

c) *Outcome ascertainment for those who leave the health system*. Attrition rates from the UPMC health plan and PCP practices are ~3-5%/year. These rates reflect UPMC's dominant position in the west Pennsylvania market (i.e., 2.9 million patients, 60% market share in Allegheny County; 41% market share over 29 counties and >500 provider offices). We have accounted for these attrition estimates in our power calculation and our statistical approach is well suited to account for data missing at random.

d) *Surveillance bias*: Patients with high-risk CKD usually receive frequent lab monitoring, often immediately preceding or following their PCP visits. However, patients in the intervention group could receive additional monitoring under the guidance of a nephrologist, potentially causing an increased likelihood of achieving a lab based outcome event (e.g., eGFR decline). Our preliminary data suggests this will be limited as 75% of high-risk CKD patients have at least 1 eGFR/serum creatinine every 6 months and over 90% have at least 1 BP measure every 6 months. However, to minimize this bias, we are: a) *using an annual decision support alert to remind study PCPs in both arms to order a BMP on study patients, if results are not available in the last 6 months, b*) using an eGFR decline of 40%, which was robust to type I errors,[92] c) modeling individual eGFR trajectories with splines to determine the time to event, and d) conducting sensitivity analyses requiring 2 consecutive eGFR values below threshold.

e) *Lack of ascertainment of OTC NSAIDs in the usual care arm*: The intervention arm may have higher rates of documented NSAID use related to ascertainment of OTC use during medication reconciliation. Because the usual care group will not receive medication reconciliations, some OTC NSAID use is likely to be misclassified. We will take several approaches to address this. First, we will compare NSAID use between arms based on prescription use only. This will be ascertained from the EHR and using administrative data from the health plan. Notably, baseline prescription use is ~20% in this high-risk group. Second, in an exploratory analysis, we
