# Supplementary material for: Electronic Health Record Population Health Management for Chronic Kidney Disease Care: A Cluster Randomized Clinical Trial
Source: JAMA Intern Med. 2024 Apr 15;184(7):737–47. doi: 10.1001/jamainternmed.2024.0708 (PMC11019443; doi:10.1001/jamainternmed.2024.0708)
Supplement: Supplement 4. — eTable 1. Baseline characteristics of PCP practices by intervention arm eTable 2. Treatment fidelity in intervention arm eTable 3. Effect of intervention on primary outcome stratified by race eTable 4. Effect of intervention on albuminuria (using fitted a mixed effects model adjusted for age, sex, race, baseline eGFR, practice size and diabetes) eTable 5. Adverse events by intervention arm [file jamainternmed-e240708-s004.pdf]

Supplemental Online Content

Jhamb M, Weltman MR, Devaraj SM, et al. Electronic health record population health management for chronic kidney disease care: a cluster randomized clinical trial. *JAMA Intern Med*. Published online April 15, 2024. doi:10.1001/jamainternmed.2024.0708

eTable 1. Baseline characteristics of PCP practices by intervention arm

eTable 2. Treatment fidelity in intervention arm

eTable 3. Effect of intervention on primary outcome stratified by race

eTable 4. Effect of intervention on albuminuria (using fitted a mixed effects model adjusted for age, sex, race, baseline eGFR, practice size and diabetes)

eTable 5. Adverse events by intervention arm

This supplemental material has been provided by the authors to give readers additional information about their work.

© 2024 Jhamb M et al. *JAMA Internal Medicine.*

eTable 1. Baseline characteristics of PCP practices by intervention arm

**Variable**	**Overall (N=98)** Mean (SD) or n (%)	**Control Group (N=50)** Mean (SD) or n (%)	**Intervention Group (N=48)** Mean (SD) or n (%)	**Absolute Standardized Bias**
Number of Locations				
1	70 (71.4%)	36 (72.0%)	34 (70.8%)	2.58
2	14 (14.3%)	5 (10.0%)	9 (18.8%)	25.14
3	8 (8.2%)	4 (8.0%)	4 (8.3%)	1.22
4+	6 (6.1%)	5 (10.0%)	1 (2.1%)	33.70
Number of Physicians per practice	4.2 (4.3)	4.2 (3.5)	4.2 (5.0)	0.78
Number of Family Practice physicians per practice	2.8 (4.3)	3.0 (3.6)	2.6 (5.0)	8.85
Number of Internal medicine physicians per practice	1.8 (2.3)	1.8 (2.4)	1.8 (2.3)	1.63
Physicians with International Medical Graduate Degree per practice	1.0 (1.3)	1.2 (1.5)	0.8 (1.1)	27.70
Physicians with US-MD Degree per practice	2.5 (3.6)	2.5 (2.4)	2.6 (4.5)	4.47
Physicians with US-DO Degree per practice	1.1 (1.2)	1.3 (1.1)	1.0 (1.2)	18.36
Number of Male Practitioners per practice	2.4 (2.5)	2.4 (2.0)	2.4 (2.9)	2.15
Number of Female Practitioners per practice	2.9 (2.6)	3.0 (2.3)	2.8 (3.0)	8.81
Total Practitioners (Physicians, Physician Assistants, Nurse practitioners)	5.1 (4.7)	5.2 (3.8)	5.1 (5.5)	1.60
Practices with Any Physician Assistants or Nurse practitioners	47 (48.0%)	23 (46.0%)	24 (50.0%)	8.01
Practice Size (Number of CKD stage 4/5 patients not seeing a nephrologist)				3.39
<15	58 (59.2%)	30 (60.0%)	28 (58.3%)	
≥15	40 (40.8%)	20 (40.0%)	20 (41.7%)	

© 2024 Jhamb M et al. *JAMA Internal Medicine.*

eTable 2. Treatment fidelity in intervention arm

	Total encounters or n (%)	Total distinct patients (%)	Number of Encounters per patient Mean (SD)
E-consult	1693	734 (97.3%)	2.31 (1.15)
Medication Review	1836	731 (96.9%)	2.51 (1.12)
Phone review	814 (44.3%)		
Chart review (if patient unable to be contacted or unwilling to complete phone review)	1022 (55.7%)		
Education	876	469 (62.2%)	1.87 (0.88)

© 2024 Jhamb M et al. *JAMA Internal Medicine.*

eTable 3: Effect of intervention on primary outcome stratified by race

		HR (95%% CI)
Race	White	1.83 (0.86, 3.88)
	Others	0.80 (0.53, 1.21)

*P-value for heterogeneity of treatment effects = 0.06

Patients with Black and other races reported in Table 1 were combined as “Others” for this analysis

© 2024 Jhamb M et al. *JAMA Internal Medicine.*

eTable 4: Effect of intervention on albuminuria (using fitted a mixed effects model adjusted for age, sex, race, baseline eGFR, practice size and diabetes)*

Endpoint	Kidney CHAMP	Control	Kidney CHAMP vs Control Mean Difference (95% CI)	P
UACR, change from baseline to 18mos[$]	22.9 (-46.5, 92.4)	41.2 (-30.0, 112.3)	-18.2 (-117.6, 81.2)	0.72

*All patients with at least 1 UACR measure during the study period were included. There were 1524 of 1596 (96%) patients included in the analyses, with 1451 (91%) having baseline, 1188 (74%) having follow-up, 1115 (70%) having both baseline and follow-up UACR.

[$] Timepoint 18 months chosen as it approximated the median follow-up time in the study. Changes in UACR from baseline to 18 months were calculated by group, and between group differences were compared using contrasts

© 2024 Jhamb M et al. *JAMA Internal Medicine.*

eTable 5. Adverse events by intervention arm

AE/SAE	Overall		Control		Kidney CHAMP	
	Patients	Events	Patients	Events	Patients	Events
	(N=1,596)<br>n (%)		(N=842)<br>n (%)		(N=754)<br>n (%)	
Moderate hyperkalemia (Serum potassium 5.5 – 6 meQ/L)	136 (8.5%)	194	77 (9.1%)	107	59 (7.8%)	87
Severe hyperkalemia (Serum Potassium > 6.0 meQ/L)	39 (2.4%)	51	21 (2.5%)	28	18 (2.4%)	23
ED/hospitalizations	857 (53.7%)	2,146	449 (53.3%)	1,134	409 (54.2%)	1,020
Deaths	272 (17 %)	272	130 (15.4%)	130	142 (18.8%)	142

*p-values not reported as we did not adjust for multiplicity in analyses

**AE: Adverse Events; SAE: Serious adverse events; ED: Emergency department visit; AEs/SAEs were included until primary endpoint or competing event and deaths were included until end of study (July 31, 2022)

© 2024 Jhamb M et al. *JAMA Internal Medicine.*
