# Supplementary material for: Electronic Health Record Population Health Management for Chronic Kidney Disease Care: A Cluster Randomized Clinical Trial
Source: JAMA Intern Med. 2024 Apr 15;184(7):737–47. doi: 10.1001/jamainternmed.2024.0708 (PMC11019443; doi:10.1001/jamainternmed.2024.0708)
Supplement: Supplement 5. — Data sharing statement [file jamainternmed-e240708-s005.pdf]

Data Sharing Statement

Data
Data available: Yes
Data types: Deidentified participant data
How to access data: contact Manisha Jhamb; e-mail, jhambm@upmc.edu.
When available: beginning date: 01-01-2026, end date: 12-31-2027

Supporting Documents
Document types: None

Additional Information
Who can access the data: These data will be made available to investigators with a methodologically sound proposal, with institutional review board approval and demonstration of resources to be able to undertake the proposed analyses, for a wide range of purposes subject to review and approval by the study's executive committee, and after approval of the proposal, consistent with guidelines of the University of Pittsburgh.
Types of analyses: as above
Mechanisms of data availability: as above
